# Molecular Detection and Differentiation of Arthropod, Fungal, Protozoan, Bacterial and Viral Pathogens of Honeybees

**DOI:** 10.3390/vetsci9050221

**Published:** 2022-05-02

**Authors:** Lucas Lannutti, Fernanda Noemi Gonzales, Maria José Dus Santos, Mónica Florin-Christensen, Leonhard Schnittger

**Affiliations:** 1 Instituto de Patobiología Veterinaria (IPVET), Instituto Nacional de Tecnología Agropecuaria-Consejo Nacional de Investigaciones Científicas y Tecnológicas (INTA-CONICET), Hurlingham 1686, Argentina; lannutti.lucas@inta.gob.ar (L.L.); jacobsen.monica@inta.gob.ar (M.F.-C.); 2 Escuela Superior de Ciencias Exactas, Universidad de Morón (UM), Morón 1708, Argentina; 3 Consejo Nacional de Investigaciones Científicas y Tecnológicas (CONICET), Buenos Aires 1917, Argentina; dussantos.maria@inta.gob.ar; 4 Instituto de Virología e Innovaciones Tecnológicas (IVIT), Instituto Nacional de Tecnología Agropecuaria-Consejo Nacional de Investigaciones Científicas y Tecnológicas (INTA-CONICET), Hurlingham 1686, Argentina; gonzalez.fernanda@inta.gob.ar

**Keywords:** *Apis mellifera*, apiculture, beehive, bee pathogens, varroosis, nosemosis, bee viruses, molecular detection, molecular assays, genotypification

## Abstract

The honeybee *Apis mellifera* is highly appreciated worldwide because of its products, but also as it is a pollinator of crops and wild plants. The beehive is vulnerable to infections due to arthropods, fungi, protozoa, bacteria and/or viruses that manage to by-pass the individual and social immune mechanisms of bees. Due to the close proximity of bees in the beehive and their foraging habits, infections easily spread within and between beehives. Moreover, international trade of bees has caused the global spread of infections, several of which result in significant losses for apiculture. Only in a few cases can infections be diagnosed with the naked eye, by direct observation of the pathogen in the case of some arthropods, or by pathogen-associated distinctive traits. Development of molecular methods based on the amplification and analysis of one or more genes or genomic segments has brought significant progress to the study of bee pathogens, allowing for: (i) the precise and sensitive identification of the infectious agent; (ii) the analysis of co-infections; (iii) the description of novel species; (iv) associations between geno- and pheno-types and (v) population structure studies. Sequencing of bee pathogen genomes has allowed for the identification of new molecular targets and the development of specific genotypification strategies.

## 1. Introduction

The Western honeybee (*Apis mellifera*) is of enormous economic and cultural relevance to human society. On the one hand, its service as a pollinator of crops is indispensable to procuring food production worldwide, and on the other hand, it provides subsistence for beekeepers and farmers, and apicultural activity is associated with important social and cultural values. In addition, honey commercialization and export is a source of income for several countries around the world, and constitutes a natural food with many potential health qualities [1,2].

**Figure 1:**
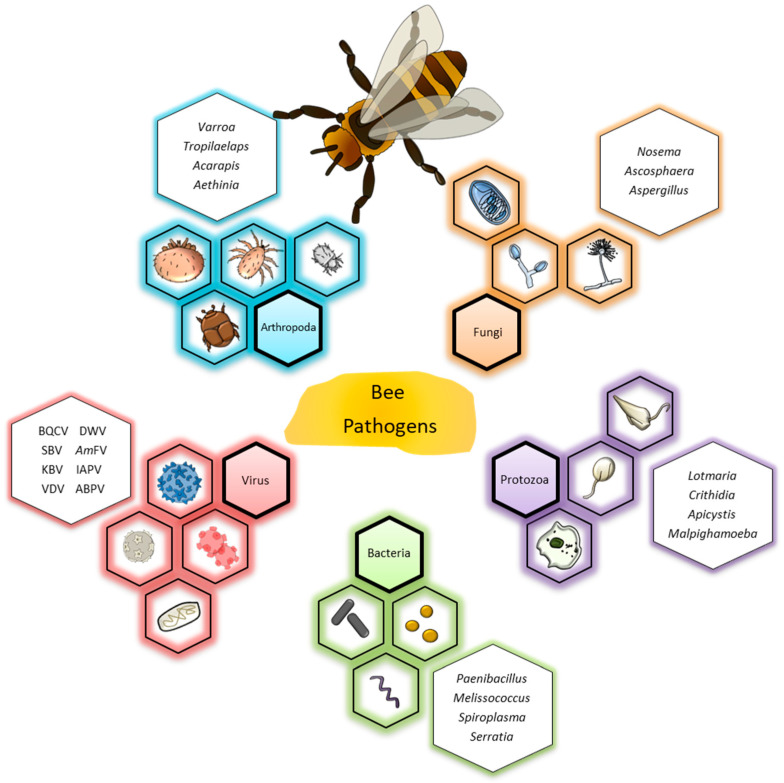
Main bee pathogens. Schematic representation of the main pathogens affecting *Apis mellifera*, belonging to the Arthropoda, fungi, protozoa, bacteria and virus taxonomic groups.

Increasingly, managed honeybee colonies face a number of challenges. One of these is their potential susceptibility to a large variety of pathogens belonging to each of the following organism groups: Arthropoda, fungi, protozoa, bacteria, and viruses [3] (Figure 1). A honeybee colony is characterized by particular features such as warmth and humidity and a large number of different ecological niches that are attractive to infectious agents, such as the different bee castes (drones, workers and queens) and developmental stages (e.g., eggs, larvae, pupae and adult bees), each of which represents a potential pathogen target [4]. Finally, honeybee foraging activity represents a possible risk of pathogen transfer to and from other pollinators when gathering pollen and nectar [5,6]. Noteworthy, different pathogens often simultaneously infect the beehive, further decreasing colony health and making it vulnerable to other challenges.

In this scenario, humans play an important role in facilitating and promoting the spread of honeybee pathogens. Colonies are managed in high densities that promote spill over infections between hives and from wild to managed pollinators [6,7]. Most importantly, the brisk international trade of beekeeping products (e.g., honey, pollen) and honeybees (e.g., queen bees, sperm) facilitates and promotes the spread of pathogens, some of which are now firmly established worldwide [8]. This is the case of the bee-infesting arthropod *Varroa destructor*, and of the fungal microsporidium *Nosema ceranae* [9,10]. Although bees have an impressive number of sophisticated possibilities in terms of innate and social immunity allowing them to defend themselves against pathogens in their natural habitat, they are not well-adapted to these exotic pathogens [11].

In order to face these challenges, a growing number of powerful molecular assays have been developed in recent decades. These tools enable and facilitate the detection of bee pathogens, allowing us to differentiate with high specificity and sensitivity diverse species and their genotypes. In some cases, molecular tools have been imperative for the recognition of novel, previously unrecognized species. For example, the arthropod species *V. destructor* could be distinguished from *V. jacobini*, and the bee-infecting protozoon *Crithidia mellificae* was found to represent two species, one of which was named *Lotmaria passim* [12,13]. Furthermore, molecular tools for the differentiation between frequently coinfecting species such as the microsporidia *N. apis* and *N. ceranae* have been developed [14,15]. Of note, based on molecular phylogenetic analysis, reclassification of both *Nosema* spp. into *Vairimorpha* spp. was recently proposed [16].

In addition to the well-established DNA amplification methods of PCR and its variants (nested and seminested PCR, multiplex PCR, qPCR, PCR-RFLP), loop-mediated isothermal amplification (LAMP) assays have been developed for the detection of several bee pathogens (for list of abbreviations see Table S4). The latter have the advantages of ease, low-cost, speed and the lack of a need for special equipment such as a thermocyclers or electrophoresis devices, since reactions take place under isothermal conditions, and results can be, under some conditions, observed by the naked eye [17]. When several DNA detection methods are available, the choice of experimental strategy should respond to the special needs of the planned investigation, the nature of the pathogen and type of sample, budget and laboratory facilities and inherent characteristics of each method (Table S1).

For some pathogens, molecular tools have allowed for unravelling the diversity and structure of their population. This permits us to estimate the approximate time and origin of the dissemination of some arthropod and fungal pathogen species that are now distributed worldwide and are of particular concern for beekeepers, such as *V. destructor* and *N. ceranae*, respectively [9,10,18].

The recent developments of molecular typing systems for some bacterial pathogens, such as multilocus sequence typing (MLST) and its related techniques, allows for the detection of the source and transmission route of outbreaks (e.g., *Paenibacillus larvae* by stable wgMLST, *Melissococus plutonius* by MLST), as well as the worldwide surveillance of strains [19,20,21]. Finally, molecular assays that allow for simultaneously detecting and quantifying a large number of virus species have been developed [22,23]. In this context, it should be noted that coinfection of certain viruses with *V. destructor* results in a particularly pathogenic course of an infection [24]. In order to orient researchers newly approaching the field of molecular genotyping, the principal strengths and weaknesses of each typification method are presented (Table S2).

**Figure 2:**
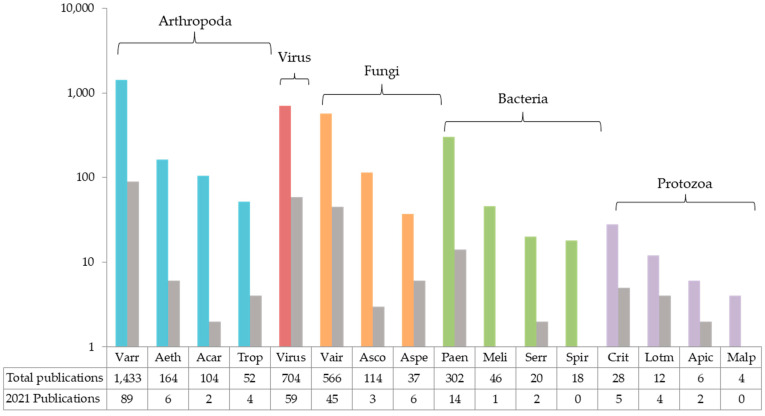
Total number of publications per pathogen type. Measurement of the research activity on bee pathogens, represented by the sum of publications per genus of the main studied organisms that affect *A. mellifera*. The total number of publications from 1979 to January 2022 and the actual number of publications released in 2021 are shown as an indication of the long-term and actual interest in these pathogens, respectively. Searches were carried out in Scopus^®^ using “*Apis mellifera*” and, with the exception of viruses, the corresponding genus as keywords, e.g., “*Varroa*”. The number of publications released in 2021 for each pathogen is represented by darker colored columns besides the corresponding column. The genera are grouped by kingdom. Abbreviations: *Varroa* (Varr)*, Aethina* (Aeth), *Acarapis* (Acar), *Trophilaelaps* (Trop), *Nosema* (Nose), *Ascosphaera* (Asco), *Aspergillus* (Aspe), *Paenibacillus* (Paen), *Melissococcus* (Meli), *Serratia* (Serr), *Spiroplasma* (Spir), *Frishella* (Fris), *Crithidia* (Crit), *Lotmaria* (Lotm), *Apicystis* (Apic), *Malpighamoeba* (Malp). All bee-infecting viruses are considered together. Scale: log_10_. More information can be found in Table S3.

The research activity, as indicated by the number of publications that appeared in 2021, is focused predominantly on *Varroa* (*n* = 89), followed by viruses (*n* = 59), and *Nosema* (*n* = 45), while considerably lesser research activity is seen for bacterial (*n* = 17) and protozoan pathogens (*n* = 11). These tendencies are also observed when all publications from 1979 until present are taken into account, and this probably reflects the assumed importance for bee health and the economy of each type of pathogen (Figure 2).

In this review, available molecular assays to detect, differentiate, and genotype the principal arthropods, fungi, protozoa, bacteria, and virus pathogens of the Western honeybee are presented and discussed. This overview will help to (i) identify research needs for the development of additionally required molecular diagnostics and (ii) select the most adequate molecular tools for future research projects.

## 2. Description of Main Bee Pathogens and Molecular Methods for Their Detection

### 2.1. Arthropoda

#### 2.1.1. Overview

Three groups of mites and one insect species are economically relevant arthropods infesting honeybees. The most notorious threat to hives is represented by the *Varroa* genus, with the worldwide distributed *V. destructor* and the Asia-confined *V. jacobsoni* [10]. Additional mite genera affecting bee health are the tracheal colonizer *Acarapis woodi* and the ectoparasites *Tropilaelaps mercedesae* and *T. clareae*. In general, mites feed on bees, weakening their immunity and lowering their performance, thus ultimately compromising the stability of the hive [25,26]. Finally, the opportunistic parasitic insect, *Aethina tumida*, better known as the small hive beetle (SHB), is from a global perspective considered a minor pest that feeds on larvae and bee products and uses the hive as a nest. However, as an invasive species, this insect has generated great losses in some geographic regions and its further distribution is of considerable concern [3]. Arthropods also act as vectors of highly pathogenic viruses, such as DWV, facilitating and promoting their distribution. With the exception of *A. woodi*, arthropod pathogens are easily recognized with the naked eye. Thus, DNA-based molecular techniques are mainly used to classify and recognize arthropod species and to study intraspecific variation. This section reviews the most important arthropod pathogens and provides an overview of the currently known molecular assays for their interspecific and intraspecific detection and/or differentiation.

#### 2.1.2. *Varroa destructor* and *V. jacobsoni*, Causative Agents of Varoosis

Varroosis is considered the main pest threatening beekeeping around the world, as supported by the estimated number of associated beehive losses and reflected by a large body of scientific publications (Figure 2) [27,28,29,30,31,32,33]. It is caused by obligate ectoparasitic mites of the family Varroidae, genus *Varroa*. The genus comprises four species (*V. destructor*, *V. jacobsoni*, *V. rindereri*, *V. underwoodi*) of which *V. destructor* [12] and *V. jacobsoni* [34] are the most important [35]. Molecular species identification by Anderson and Trueman [12] established *V. destructor* as the principal mite successfully colonizing *A. mellifera* beehives, while *V. jacobsoni* mainly infects *A. cerana*, and is therefore less relevant for apiculture. Importantly, all publications before the year 2000 need to be interpreted with caution since the binomen *V. jacobsoni* had been used until then for both species [12,32].

*Varroa* spp. adult female mites are reddish and round-shaped with a 1.5 mm width, while males and nymphs are smaller and white, but all developmental stages are visible with the naked eye [36]. The reproductive cycle starts with the deposition of an unfertilized haploid egg by a foundress female mite that colonized a beehive cell before capping. Due to Arrhenotokous parthenogenesis (haplodiploidy), a haploid male develops after hatching. Subsequent eggs of the foundress mite are fertilized, and diploid sister mites develop, with which the mite brother male mates. During this time, the foundress mite and its offspring alternatively feed on the larvae and defecate at the side of the cell until it is uncapped. While the male never leaves the cell, foundress and sister mites leave and colonize other cells to attack additional bee larvae, starting a new reproductive cycle. Alternatively, by infesting nursing bees that feed them, they start the subsequent dispersal phase of the infestation in the whole beehive [10,32,36]. Even though there is no fixed threshold for the number of mites per colony that correlates with hive damage, mite numbers ranging between 2000 and 4000 per colony have been shown to correlate with colony collapse [37]. Furthermore, it has been reported that hives which exceed infestation rates of 30% do not survive the winter [32,36,38,39].

It has been long-assumed that *Varroa* spp. mites feed on hemolymph, yet recent research has shown that *Varroa* mites feed primarily on the fat body tissue of bee larvae, pupae, and adult individuals, resulting in bee weight loss and a decreased life expectancy and flight performance [40,41]. In addition to the direct effects of *Varroa* spp. infection, resulting in the weakening of bees and a decline in bee colony health, the wounds caused by this ectoparasite constitute entry sites for other pathogens. Importantly, *Varroa* spp. functions as a mechanical and biological vector of several important viruses infecting honeybees, such as DWV (deformed wing virus), KBV (Kashmir bee virus), ABPV (acute bee paralytic virus), and SBPV (slow bee paralysis virus), of which the latter shows a negligible prevalence [42,43,44,45,46]. Other viruses such as SBV (sacbrood virus) are not directly transmitted by *Varroa* but act as a cofactor that influence resistance to and survival of mites [30,47,48].

Most of the viruses transmitted by *Varroa* play an important role in bee health. It is noteworthy that although these viruses are globally distributed and have a high prevalence, much of the pathogenesis attributed to them is displayed only in active *Varroa* infections [49]. Colony losses associated with varroosis are related to high numbers of mite-infested bees, development of mite resistance to acaricides, and the high vectorial capacity of mites for virus transmission. Synergistic biotic and abiotic factors that aggravate mite infestation are represented by the uncontrolled use of herbicides and biocides, excess monocultures and climate change [32,50]. 

Diagnosis of varroosis is a routine practice in bee breeding, performed by direct examination of debris, adults and/or brood [51]. Determination of the colony infestation rate takes place in a similar way around the world. The three principal approaches to estimate mite-infestation rates are (i) counting mites after hive treatment with acaricides, (ii) estimating the number of living mites based on the natural mortality as determined by dead mites found at the hive bottom, or (iii) direct estimation of mites infesting adult bees or brood cells. All three approaches produce estimates when mite infestation is medium-to-high, but only treatment with acaricides and the subsequent counting of dead mites result in good estimates when infestation is low. A widespread, non-destructive method is the sampling of adult bees from the colony and their subsequent treatment with icing sugar, which dislodges mites from bees; this allows for determining the average mite-infestation rate of bees. Importantly, sampled worker bees can be returned to their colonies where they are cleaned by their nest mates [26,36,52,53]. Sampling is typically carried out during honey harvesting and before winter; additional sampling is occasionally organized to evaluate the evolution of infestation and monitor the effectiveness of treatments with acaricides and other pesticides [36].

Multiple studies propose PCR-based methods for the molecular detection of *Varroa* spp. (Table 1) [12,54]. However, as outlined above, it is important to note that the quantification of the load of infestation is commonly carried out by direct observation and counting of mites, and thus, molecular techniques are not needed for routine diagnosis [36]. On the other hand, when species identification is required, molecular methods are important and the preferred approach to distinguish the closely related *V. destructor* and *V. jacobsoni* is by amplification of the mitochondrial *cox1* gene and posterior amplicon sequencing [12,36,55].

Additionally, molecular tools are indispensable for the study of intraspecies variation by genotypification and/or molecular epidemiology [12,54,56]. For this purpose, several marker sequences of *V. destructor* have been identified and are available in GenBank (Table 1) [10,55,57,58,59,60,61,62,63]. By PCR amplification and subsequent sequencing, different haplotypes based on mitochondrial *cox1*, *cytb*, *cox3* and *atp6* gene sequences have been defined for *V. destructor*, among which the haplotypes K and J (based on *cox1* polymorphism), originally identified in Korea and Japan, respectively, are the most relevant [10,56,62]. Navajas et al. [56] found that each of the six different groups of mites with identical *cox1* sequences identified in their study represent a haplogroup, designated with the initial letter of the country followed by a number (e.g., K1, J1, C1, C2, C3, and V1). Furthermore, mites of the same haplogroup that showed haplotype variations within concatenated *cytb*, *cox3* and *atp6* gene sequences were regarded as variants of a particular haplogroup (e.g., K1-1, K1-2, K1-3, etc.) (for more details see [10]). Accordingly, by concatenating mitochondrial haplotype sequences, 18 haplotypes of *V. destructor* could be assigned to six haplogroups of *V. destructor* infesting *A. mellifera* and/or *A. cerana*. Recently, 14 additional haplotypes and three additional haplogroups (K2, J2, and C4) were identified in *A. mellifera* and/or *A. cerana* in China [64]. Due to a bottleneck event where a host switch from *A. cerana* to *A. mellifera* occurred, some variants of haplogroups K1 and J1 are exclusively identified in *A. mellifera* (e.g., K1-1/2, K1-4, K1-5, J1-1, and J1-6). Altogether, based on available mitochondrial sequence data, it has been deduced that at least 32 haplotypes of *V. destructor* (of which 22 belong to the K type) and 19 of *V. jacobsoni* exist [10,12,54,56,65]. Access to an interactive map of the worldwide distribution of *V. destructor* haplotypes on *A. mellifera* and *A. cerana* and of *V. jacobsoni* haplotypes on *A. cerana* can be found in Supplementary Materials of Traynor et al. [10].

Additional haplotypes that have been designated S1 and P1 represent *cox1* and *cytb* polymorphisms, respectively, and have been found to be regionally confined to Serbia. Other haplotypes exclusively described in Argentina are the K sub-haplotypes KArg-N1 and KArg-N2 that represent polymorphisms of the *nd4* gene. Interestingly, of these polymorphisms, heteroplasmic variants including KS1, KP1, and KArg-N1/KArg-N2 have been identified in Serbia and Argentina, respectively [66,67,68]. The K haplotype, found in most regions of the world, is by far the most successful in terms of colonization, and has the highest reproductive indices in *A. mellifera* [10,60,69]. The haplotype J has been found to be confined to Japan, Thailand and some regions of the Americas (North America, Chile, and Brazil), but seems to be increasingly replaced by the K haplotype [10,12,60,70].

Some genotyping methods that do not require sequencing have also been described, such as PCR-RFLP (PCR restriction-fragment-length polymorphism) protocols that discriminate the haplotypes J, K and P1, based on the sizes of digested fragments [60,66,71]. The haplotypes K and S1 could also be distinguished by ARMS-PCR (amplification-refractory mutation system PCR), a PCR format in which amplification primers target variant-specific polymorphisms, which makes sequencing dispensable [66,67,72]. Recently, *Varroa* haplotypes from environmental samples (honey) were studied via sequencing of PCR amplicons. Results showed a high prevalence of the K1 haplotype, only a few samples of the J1 haplotype from South American samples and a C1 haplotype from Chinese honey samples [62].

Microsatellites present in the nuclear genome have also been targeted for genetic studies of *V. destructor,* and have demonstrated a clonal population structure corresponding to the K and J mitochondrial haplotypes as assessed by PCR-RFLP [60,73,74]. This clonal structure has been suggested to be due to a founder effect when *V. destructor* shifted from *A. cerana* to *A. mellifera* about 50 years ago. However, population admixtures and F1 hybrids are observed, suggesting that double infestations occur in some regions in a high proportion [60]. Of note, a clonal structure is also suggested by estimation of an unusually low rate of genomic SNPs (single-nucleotide polymorphisms) within the *V. destructor* population [63].

Importantly, mutations associated with resistance to biocides such as pyrethroids and tau-fluvalinate have been detected by conventional PCR, PCR-RFLP and single-strand conformation polymorphism after PCR amplification (PCR-SSPC). This indicates that molecular techniques applied to monitoring biocide tolerance in epidemiological studies provide a useful tool to optimize control of varroosis [75,76].

#### 2.1.3. *Acarapsis woodi* Causing Acarapiosis

The mites of the *Acarapsis* genus—*A. externus*, *A. dorsalis* and *A. woodi*—are obligatory bee parasites long-recognized as apicultural pests [26]. While the first two are ectoparasites present on the head and thorax, respectively, *A. woodi* is an endoparasite infecting the trachea of bees and is the most relevant due to the severe pathogenesis it causes, referred to as acarapiosis [77,78]. Specifically, *A. woodi* blocks the thoracic tracheae, reducing oxygen diffusion to flight muscles and the brain. In addition, it may act as a vector of bacterial and virus pathogens. Severe bee infestation results in a shortened bee lifespan, diminishes brood, resulting in smaller bee populations, and promotes colony failure [26]. In spite of its associated economic impact, *A. woodi* tends to be disregarded, possibly due to a general lack of awareness and the few visible signs associated with the infestation, such as weakened bees with crossed wings forming a K [26,79].

Classical morphological diagnostic methods are based on the dissection of the thoracic disc of individual bees (thoracic disc method) followed by laborious and time-consuming microscopic examination. For this reason, molecular detection methods have been developed that facilitate routine screening [80,81]. Furthermore, ELISAs based on specific antisera against *A. woodi* can detect infestations with higher than 5% prevalence [82,83]. As of now, all molecular diagnostic assays for *Acarapsis* spp. detection use the mitochondrial *cox1* gene as a target (Table 1) [80,84,85,86,87]. However, some of these molecular assays lack specificity and may amplify, besides *A. woodi*, *A. externis* and/or *A. dorsalis* DNA. Thus, subsequent amplicon sequencing for correct estimation of the prevalence of *A. woodi* is needed [80,84]. Interestingly, the approach of amplicon sequencing allowed for identifying a *A. woodi* variant representing a putative novel species [86,88]. Importantly, specific amplification of *A. woodi* DNA, discriminating this species from *A. externis,* has been achieved by conventional PCR assay, and distinction of *A. woodi* from *A. externis/A. dorsalis* could be achieved by qPCR [86,87]. Furthermore, using a conventional PCR format based on amplification primers targeting *cox1* designed by Kojima et al. [85], Garrido-Bailon et al. [80] and Ribani et al. [89] achieved detection of *A. woodi*-specific DNA in honey samples. It would be desirable to develop additional species-specific molecular diagnostics for each of the three recognized species of *Acarapsis*.

#### 2.1.4. *Tropilaelaps mercedesae* and *T. clareae*

*Tropilaelaps* sp. are bee-specific mites distributed exclusively throughout the Asia region, with a similar behavior and life cycle to *Varroa* sp., but of smaller size [25,89,90]. There are four described species of *Tropilaelaps* sp.: *T. thaii*, *T. koenigerum*, *T. clareae* and *T. mercedesae*, of which *T. clareae* and *T. mercedesae* have been found to infest *A. mellifera*, the latter showing the largest geographical distribution within Asia [35,91,92,93,94]. In contrast to *Varroa* spp., *Tropilaelaps* spp. acari infest and feed exclusively on bee larvae, resulting in lower emergence weight of larvae and reduced longevity [35,95]. In addition, infestation with *Tropilaelaps* spp. promotes infection of bees with DWV, possibly by functioning as a viral vector [95,96].

As a potentially emerging threat to the European honeybee, infestation with *Tropilaelaps* spp. is a regulated disease listed by the International Office of Epizooties (OIE), and any detection of the parasite must be notified to the competent authorities. In the EU and US, regulatory measures have been implemented to avoid introducing this parasite, which would be potentially damaging for Western apiculture. Microscopic examination allows for discriminating between *Tropilaelaps* spp. and *Varroa* spp., but does not allow for determining the infecting *Tropilaelaps* species [90,97]. Using morphological methods, the four species cannot be routinely distinguished, as identification is time-consuming and requires highly experienced personnel. On the other hand, the integrity of adult samples may be compromised, and morphological methods are not applicable to immature stages. To overcome these limitations, molecular methods have been developed to facilitate species diagnosis (Table 1).

Molecular differentiation between *T. clareae* and *T. koenigerum* has been achieved by a randomly amplified polymorphic DNA (RAPD) assay, and by a PCR-RFLP assay based on polymorphism in the ITS (internal transcribed spacer) region [98,99]. In addition, molecular differentiation of all four *Tropilaelaps* species has been carried out by conventional PCR targeting the *cox1* gene and the complete ITS region followed by sequencing or RFLP analysis of amplicons [93,98,100]. In addition, high-resolution melting (HRM) analysis using a variable region of the *cox1* gene as a barcode (Bar-HRM) successfully differentiates the four *Tropilaelaps* spp. This method is based on the different melting curves of variable species-specific sequences (barcodes) obtained after PCR amplification [101].

#### 2.1.5. *Aethina tumida* or Small Hive Beetle (SHB)

*Aethina tumida*, better known as small hive beetle (SHB), is an insect of the Coleoptera order, Nitidulidae family, from sub-Saharan Africa, first described in 1867 [102,103]. Adult beetles are oval-shaped, winged, of 5–7 mm length and 3–4.5 mm width, with a reddish-brown to black color [104]. SHB lays its eggs inside the hive, and the hatched larvae feed on bee larvae and products. Its pupae stage is found on the floor surrounding the beehive, and once formed, adults search for another hive where they repeat the cycle [104,105]. In principle, SHB is considered a minor pest in its native range of distribution. Considered an opportunistic parasite, infestations are associated with weakened or poorly managed hives that cannot deal with pathogen attacks, whereas in strong hives, bees can defend themselves by the imprisonment of the beetle in propolis [104,105,106,107]. However, SHB introductions and subsequent invasions have been reported in a number of continents and countries including the USA, EU (e.g., Portugal, Italy), and Australia [105,108,109]. In these new ranges, the beetle can be a harmful parasite of colonies of European honeybee subspecies and can cause substantial losses, explaining why SHB is a notifiable pest in the EU [109]. More recently, in at least two Asian countries, invasion of SHB of the Western honeybee (Philipines) and of the Eastern honeybee (Philippines, China) were reported to cause devastating infestations [110,111]. Associated economic losses differ among countries, hive-managing strategies and climate conditions [107,112]. Notably, by application of molecular detection methods it was found only recently that SHB is a potential vector of important bee pathogens, including the fungus *N. ceranae*, the protozoans *Lotmaria passim* and *Crithidia mellificae,* and viruses DWV, KBV and AmFV (*A. mellifera* filamentous virus) [113,114,115].

Adult SHBs are notoriously difficult to detect by direct visual examination of the hive. Furthermore, as these beetles are highly migratory insects, they may have departed before hive inspection. However, SHB infestation can be suspected by brood damage or recognized by the presence of the beetle larvae on and in combs, though infestation at initial stages or at low levels may escape detection [104,109].

Importantly, detection of SHB-specific DNA in hive debris using molecular diagnostics significantly improves specificity and sensitivity of diagnosis and is either recommended or mandatory in some countries to screen hives or packaged bees before importation [116,117]. For this purpose, a number of conventional and quantitative PCR protocols based on the *cox1* gene have been designed (Table 1) [116,117,118,119]. Furthermore, a multiplex PCR format against a SHB-specific *cox1* and 18S rRNA gene region as an internal control has been established and proved to be reliable [117]. Finally, a SHB-specific *cox1* and an invertebrate control 28S rRNA gene region were used for the development of two LAMP assays, respectively, which provide a particularly fast and cost-effective detection strategy [120]. In addition to *cox1* regions for SHB detection, the invasion of SHB in the Americas has been mapped using nuclear microsatellite loci [121,122].

Of note, sequencing of the *A. tumida* nuclear and mitochondrial genome has allowed for identifying several sequences and proteins of interest to further develop and improve molecular tools for diagnostics, molecular epidemiological studies, and treatment and/or control [123,124].

**Table 1:** Molecular diagnostics for detection and inter- and intra-species differentiation of arthropods pathogenic for the honeybee.

Type of Reaction	Species or Genotypes	Target	Size of Amplicon or RFLP Pattern (nt) or Number of Markers	Accession Number	Ref.
*Varroa*					
RAPD	*V. destructor* ^a^/*V. jacobsoni*	fingerprints	variable	n.a.	[125]
PCR-amplicon sequencing	*V. destructor* *V. jacobsoni* *V. rindereri* *V. underwoodi*	*cox1*	458	AJ493124	[12]
	*V. destructor* haplogroups: K1, V1, C1, C2, C3, J1	*cox1*	929		[56]
	haplotypes: K1-1/2,3,4; V1-1,2,3,4; C1-1,2; C2-1; C3-1; J1-1,2,3,4,5,6	*atp6-cox3*	818		
		*cytb*	985		
	*V. destructor* haplogroups: K1, K2, J2, C1, C4	*cox1*	821		[64]
	haplotypes: K1-1/2,3,5,6,7,8,9,10,11,12,13,14,15; K2-1; J2-1; C1-2,C4-1	*cytb*	985		
	*V. destructor* haplogroups: K1, J1, C1-1	*cox1* *cytb* *cox3*	various	AJ493124 AY163547	[62]
	*V. destructor* haplotypes: KArg-N1, KArg-N2 heteroplasmy KArg-N1/N2	ND4-ND4L	839		[68]
ARMS-PCR	*V. destructor* haplotype: K, S1; heteroplasmy: KS1	*cox1*	variable	AF106899	[66]
PCR-RFLP	*V. destructor* haplotype J and K	*cox1*	68,129 and 273 for haplotype J; 129,341 for haplotype K (*EcoN*I) 230 and 340 for J haplotype; no cut in K haplotype (*Sac*I)	AJ493124	[71]
	*V. destructor* haplotype J and K	*cytb*	128/124 and 252/256 for J haplotype, 376 for K haplotype (*Sac*I)	AJ493124 AJ784872	[60]
	*V. destructor* haplotype K and P1 heteroplasmy: KP1		226 for haplotype K; 58,168 for haplotype P1 (*Vsp*I)	JX970945	[66]
	*V. destructor* tau-fluvalinate resistant vs. non-resistant variant	NaVCh	603 for Tau-fluvalinate resistant; 270,333 for Tau-fluvalinate susceptible homozygotes (*Sac*I)	KC152656	[76]
Microsatellites	*V. destructor* multilocus genotypes	tandem repeats	20 markers	AF229974-77 AF229979-85	[73]
			16 and 13 markers	AJ558164-79	[60,126]
			4 markers	several	[118]
			several potential markers	several	[63]
*Acarapis*					
PCR	*A* *. externus* *A* *. dorsalis* *A* *. woodi*	*cox1*	377	GQ916565	[84]
			247	AB638409 AB638410	[85]
			162	EU190886 FJ603294 FJ603296 GQ916565	[80]
	*A. woodi*, not *A. exernus*; *A. dorsalis* not confirmed		180	AB634837	[86]
qPCR	*A. woodi*	*cox1*	113	EU190886	[87]
		ITS2	94	HQ25966-7 HQ259670 FJ603297 FJ603298	[87]
*Tropilaelaps*					
RAPD	*T. clareae, T. koenigerum*	fingerprints	variable	n.a.	[99]
PCR-RFLP	*T. clareae* *T. mercedesae* *T. thaii* *T. koenigerum*	ITS	63,260,280 or 140,150,310 for *T. clareae*; 270,330 or 285,310 for *T. koemigerum* (*Mse*I and *Sau3A*I)	n.a. (universal primers [98])	[99]
		ITS1-5.8S-ITS2	distinguishes the 4 species (*Bme1580*I, *Psi*I, and *Rsa*I)	n.a. (universal primers [98])	[93]
		*cox1*	distinguishes the 4 species (*Fau*I, *Bsr*I, *BstY*I and *Swa*I)	n.a. (universal primers [100])	
qPCR	*T. clareae*	*cox1*	various	msa ^b^	[101]
*Aethina*					
PCR	*A. tumida*	*cox1*	1080	KT380626	[118]
qPCR			109	AF227645-54 AF522354–58	[116]
			396	KT380625-6 AF227647	[117]
LAMP			variable	msa ^b^	[120]
Microsatellites		tandem repeats	15 markers	several	[121]

n.a., not applicable; ^a^ Reported as *V. jacobsoni* when *V. destructor* had not yet been described; ^b^ msa: primers designed out of a multiple sequence alignment.

### 2.2. Fungi

#### 2.2.1. Overview

Insects and fungi coexist in all kinds of ecological situations, displaying different symbiotic interactions from mutualism to antagonism. Most or all insects can be attacked by facultative or obligate enthomopathogenic fungi, which use insects as a source of nutrition. These fungi manage to counteract the immune and behavioral defenses of insects through complex mechanisms that include blocking the action of immune response effectors by proteolytic cleavage of toxins or by RNA interference of the expression of host immune genes [127]. A characteristic of fungi that guarantees their success is the production of environmentally resistant spores that disseminate and infect new hosts, thus continuing species propagation. The harmful effects of some enthomopathogenic fungi has positioned them as attractive tools for the biological control of pests relevant to agriculture [128]. In the case of bees, three main genera of fungi can be harmful and are thus economically relevant for apiculture: the microsporidians *Nosema* spp. and the ascomycetes *Ascosphaera apis* and *Aspergillus* sp., causative agents of nosemosis, chalkbrood and stonebrood diseases, respectively [15,129].

#### 2.2.2. *Nosema apis* and *N. ceranae*, Causative Agents of Nosemosis

Bee nosemosis has consistently attracted the attention of beekeepers and scientists since it was reported 15 years ago that *Nosema ceranae*, considered an exclusive parasite of the Asian bee, switched host to the European bee, and was associated with beehive damage [130,131,132,133]. Considering the number of publications on nosemosis produced since then, *Nosema* spp. can be certainly placed among the most-studied pathogens of *A. mellifera* (Figure 2).

*Nosema* spp. are lower eukaryotes of the Microsporidia phylum within the Fungi kingdom [134,135], which were recently proposed to be reclassified into *Vairimorpha* spp [16]. Microsporidia are obligate intracellular parasites, have highly compact genomes and show extensive loss of genes of several metabolic pathways, indicative of a strong host dependency [136].

Two *Nosema* spp. are well-known bee pathogens: *N. apis*, identified in *A. mellifera* in Europe in 1909, and *N. ceranae*, identified infecting the Asian bee, *A. cerana*, in 1996; and ten years later reported to infect *A. mellifera* in Spain and Taiwan [130,133,137,138]. Interestingly, the finding of *N. ceranae* in preserved samples of *A. mellifera* from the 1990’s indicates that this species has been propagating among European bees for a considerable time before this was first discovered [132,139,140,141,142]. The increasing interest on *N. ceranae* research observed over the last two decades is due to several facts: (i) this species is distributed worldwide [9]; (ii) it is the main, and often the only, *Nosema* species detected in *A. mellifera* [143,144,145,146,147,148,149]; (iii) it has been associated with beehive weakening and increased deaths, especially in cases of mixed infections [9,150,151]; and (iv) infections are asymptomatic until they are highly advanced, and have a strong seasonal component [15,152,153]. The distribution of *N. apis* infections of *A. mellifera*, on the other hand, is geographically confined and erratic, and has been suggested to depend on climatic, genetic, ecological and apiary management variables [150,152,153,154,155]. *N. neumanni*, genetically close to *N. apis*, was recently described infecting the European bee in Uganda and later detected in Japan, but information on this *Nosema* species is scarce [156,157].

Nosemosis is essentially an infection of the bee gut. Spores represent the infective stage of *Nosema* sp. and are the only stage that can live outside a host cell. They contain a sporoplasm with nuclei, the polar tube—an invasion organelle that is coiled up within the interior of the spore—and a chitin-containing wall. In the bee gut, the polar tube is ejected and pierces an intestinal epithelial cell, and the sporoplasm is injected through it into the host cell. Several intracellular asexual reproduction cycles lead to the formation of primary autoinfective and secondary environmental spores, which are released to the gut lumen when the epithelial host cells are lysed. The former invades other gut cells whereas the latter are released into the environment with the feces, disseminating the infection. Transmission of spores mainly occurs through ingestion of contaminated food, and during trophallaxis and grooming [158]. A sexual route of infection has also been suggested since spores have been found in male bee sperm [159]. In addition, evidence has been presented that the SHB can be infected by *N. ceranae* and may disseminate this pathogen as mechanical and/or biological vector [115]. *Nosema* spp. parasites compete with the host for energy and nutrients, and cause lesions in gut cells, leading to an immune inflammatory response [160]. This, in combination with environmental stress and the presence of other pathogens such as *L. passim*, *C. mellificae* and the deformed-wing virus (DWV), can cause the weakening of bees, and in the long run, of the whole beehive [161,162,163,164].

Diarrhea and high winter mortality have been associated with *N. apis* infections in *A. mellifera*. *N. ceranae* infections, on the other hand, may progress in the absence of clinical signs, preventing an early diagnosis, but are also associated with high mortality, mostly in autumn and spring [15,165].

Diagnosis of nosemosis is commonly carried out by estimation of the average number of spores per bee upon microscopic observation of squashed whole bees, abdomens or guts [15]. Lower spore loads are relatively well-tolerated, but loads of over 10^6^ spores per bee have been associated with advanced and/or severe infections, leading to the decline of bee colony health, and this is considered the treatment threshold [153]. A recently developed portable device that detects chitin-positive spores by fluorescence and is coupled to a smartphone application can facilitate spore quantification, allowing for the detection of high *Nosema* sp. bee infections (>0.5 × 10^6^ spores per bee) in a given colony in the field [166].

Spore quantification methods provide an approximate idea of the seriousness of a *Nosema* spp. infection, which aids in making a decision on the need for treatment. However, they cannot discriminate between species, since *N. apis* and *N. ceranae* spores are minimally different from each other in size and shape. Molecular methods are thus favored for epidemiological studies since they are species-specific and have an increased sensitivity, detecting in many cases in the range of 10 to 100 spores per bee for simple PCR and around 1 spore per bee for qPCR or LAMP [148,167,168]. Over 30 protocols have been developed for the molecular detection of *Nosema* spp. based on simple and multiplex PCR, qPCR, RFLP-PCR, and LAMP (Table 2) [169]. Duplex formats are more convenient than uniplex PCR or qPCR protocols when applied to epidemiological scenarios where both *N. ceranae* and *N. apis* can be found. The most used target has been the 16S rRNA gene, either by amplifying species-specific segments, or by discriminating post-amplification between species by restriction enzyme digestion or amplicon sequencing [15,130,131,170]. Incorporation of a bee-specific DNA target is convenient as a DNA extraction and amplification control [171].

Although, amplification of the multicopy 16S rRNA gene usually results in high sensitivity of detection, it has been reported that single PCR formats based on this gene may lead to an overestimation of *N. ceranae* infections and/or to contradictory results due to polymorphisms possibly generated by recombination between gene copies [14,172,173]. Contradictions observed have resulted in scrutinizing the specificity, sensitivity, and reliability of available PCR assays and in the design of further improved ones [14,168]. The sequencing of *N. ceranae* and *N. apis* genomes has allowed us to identify several additional molecular targets that have been applied in molecular diagnostics (Table 2) [174,175,176]. As an example, an *N. ceranae*/*N. apis* duplex PCR based on species-specific differences in the highly conserved *rpb1* (DNA-dependent RNA polymerase II) gene resulted in higher reliability of results than simple PCR protocols based on the 16S rRNA gene [14].

Several qPCR assays have been developed for *Nosema* spp., and have the advantage of increased sensitivity and specificity; however, this needs to be balanced against the disadvantages of the higher cost and the need of expensive equipment with respect to simple PCR formats [15,146,167,177,178,179,180,181,182,183]. In addition, some qPCR assays have been developed for the quantification of the infection based on standard curves [178,180,181,184]. However, Cilia et al. [173] demonstrated that unless a single-copy gene is used, as in the case of a protocol based on the *hsp70* gene, correlation between qPCR results and spore number is not straightforward [155,173].

In addition, three loop-based isothermal amplification (LAMP) assays have been developed for the molecular detection of *N. ceranae* and one for *N. apis* [148,185,186]. Importantly, the use of the microsporidium-specific *ptp3* (polar tube protein 3) instead of the 16S rRNA gene as target decreases the risk of cross-reactivity with DNA of other microorganisms. In addition, the use of intercalating agents such as Sybr-safe allows for the direct observation of positive and negative results under UV light, decreasing cost, time and the need of laboratory equipment [148].

A number of genotyping and genomic studies have been carried out on *N. ceranae* and some on *N. apis*, demonstrating no population differentiation worldwide with respect to the former, but a significant differentiation between parasites originating from distinct phylogenetic lineages of *A. mellifera* for the latter. These studies have been carried out using MLST, comparing patterns of nucleotide polymorphism of single-copy genes, and through determination of the frequency of SNPs in the genome of isolates originating from different geographic regions around the world [18,175,187,188]. Presented data are consistent with the notion that *N. ceranae* recently changed host, resulting in a founder effect and a subsequent population expansion, whereas in the case of *N. apis*, the revealed data agree with a long-term host–parasite relationship. Interestingly, in contrast to the observed diversity between isolates from different regions, considerable allele and nucleotide polymorphism is found within *Nosema* spp. populations originating from a single colony, and it has been proposed that this is due to tetra- or poly-ploidy [175]. Since the abovementioned applied research methods could not differentiate between isolates or strains of *Nosema* spp. of different geographic origin and are therefore only of scientific interest, they have not been included in Table 2.

#### 2.2.3. *Ascosphaera apis*, Causative Agent of Chalkbrood

*Ascosphaera apis* (*A. apis*) infects and kills the larvae of *Apis mellifera*, giving them a chalky mummified aspect, thus the name of “chalkbrood” for this bee disease. Chalkbrood was first detected in Europe in the early 1900s and now appears to have spread worldwide, aided by international trade in honeybees and the persistence of spores in the environment, which can last up to 17 years. Infections are more common during spring, and different factors including bee genetics, beehive health, pathogen strain, and environmental stressors, seem to influence the severity of the disease. Infection occurs when sexually produced ascospores present in contaminated food are ingested by larvae and germinate in their gut. The growing mycelium invades the body cavity of the larvae, resulting in their death, and continues to grow, covering the entire mummified organisms. After hyphal fusion of two opposite mating types, ascomata containing infective ascospores are produced. Released ascospores accumulate in all beehive products, such as foundation wax, pollen, and honey. This hive-contaminated material serves as a source of infection between beehives [189,190].

Diagnosis of chalkbrood is usually carried out by the observation of spongy mummified larvae in the brood chambers, or at the entrance or base of the beehive [191]. Microscopic observation of ascomata has also diagnostic value [189].

Specific molecular detection of *A. apis* infections in bee larvae is recommended for the diagnosis of infestations, especially in early or subclinical cases. The target for these assays is the ITS1-5.8S rRNA-ITS2 region, and one of these assays has been successfully applied to honey samples [89,192,193,194,195]. Importantly, two of these protocols can differentiate among the four *Ascosphaera* species that infect honeybees, of which only *A. apis* is of economic relevance [193,194]. Finally, specific primers for *A. apis* detection have been included in a multiplex PCR that also detects two bacterial diseases, American and European foulbrood [195].

Early approaches for the genotypification of *A. apis* isolates were based on amplification and sequencing of ITS1, ITS2, and the 5.8S rRNA gene or fingerprinting using rep-PCR of repetitive BOX, ERIC, and REP sequence elements that have been originally identified in bacteria (see Section 2.4.2) [100,196]. As a disadvantage, the latter method is not species-specific and thus requires the production of axenic cultures of *A. apis* prior to DNA extraction. After the publication of the *A. apis* genome in 2006, 25 microsatellite markers have been identified for genotypification not requiring prior isolation of the fungus. These markers should be useful for differentiating strains according to their virulence and for population structure studies [197,198,199].

More recent molecular investigations on *A. apis* integrated metagenomic and transcriptomic studies to analyze the composition of the bee microbiota and the interactions among microbiota microorganisms in relation to bee health [200,201,202].

#### 2.2.4. *Aspergillus* sp.

The *Aspergillus* genus has over 300 species that can colonize different insects, other animals including humans, and plants [203,204]. They are considered facultative pathogens and their pathogenicity depends on the condition of the host [129,205]. The most important *Aspergillus* species for apiculture are *A. flavus*, *A. fumigatus*, and *A. niger*. Pathogenic effects are associated with colonies that have been weakened by other factors, but the predisposing conditions as well as the contribution of each *Aspergillus* species to disease are unknown [129,206]. Prevalence of *Aspergillus* spp. in the bee microbiota is highly variable, ranging from 0.1 to 43% according to metagenomic studies [207]. In clinical cases, *Aspergillus* spp. spores germinate on the cuticle, or, when spores are ingested, in the gut, and invade bee tissues. Asexual conidia are airborne and easily disseminate the infection [205].

*Aspergillus* spp. infections lead to hard-mummified larvae, thus the name of “stonebrood” for this disease. In addition, *Aspergillus* spp. can also affect adult bees [208]. A preliminary field diagnosis based on the presence of spongy or hard-mummified larvae can be carried out to differentiate chalkbrood from stonebrood [129].

Molecular methods are rarely used for *Aspergillus* sp. in bees, but PCR protocols exist for the discrimination of ascomycetes from different fungal groups, targeting genes of taxonomic relevance [98,129,209]. Specific detection of *A. flavus* in bees could be achieved based on a LAMP protocol targeting the 18S rRNA sequence, which has been shown to be specific and rapid, and results could be directly visualized by adding a fluorescent intercalant agent to the amplification reaction [210].

**Table 2:** Molecular assays for the detection and differentiation of fungal pathogens of the honeybee.

Type of Reaction	Species	Target	Size of Amplicon (nt)	Accession Number	Ref.
*Nosema*					
PCR		16S rRNA	222	AY741110 U97150 U26533	[131]
			208,212	U97150 DQ486027	[142]
			488	U97150	[211]
*Nosema apis*					
PCR		16S rRNA	209	857487	[212]
			240	U26534	[130]
			325	U97150	[213]
			401		[142]
PCR-RFLP		16S rRNA	91,136,175 (*Msp*I, *Nde*I)	U97150	[131]
		18S rRNA	433		[214]
qPCR		16 rRNA, ITS, 18S rRNA	269	U97150	[177]
		16S rRNA	104		[167]
			278		[178]
			103		[180]
			312	DQ235446	[215] (improvement on [170])
		*rpb1*	71	DQ996230	[146]
LAMP		16S rRNA	variable	JQ639306	[185]
*Nosema ceranae*					
PCR		16S rRNA	252	U26533	[130]
			250	DQ486027	[142]
PCR-RFLP		16S rRNA	104,116,177 (*Msp*I, *Pac*I)	DQ078785	[131]
		18S rRNA	175 and 262		[214]
qPCR		16S rRNA, ITS, 18SrRNA	250	DQ486027	[177]
		16S rRNA	142		[167]
		18 rRNA	316		[178]
		16S rRNA	92		[180]
		hsp70	65	XM_002995382	[181]
		16S rRNA	221	DQ329034	[215] (improvement on [170])
		*ptp3*	90	XM_002996713	[146]
		5S rRNA, 16S rRNA, ITS	216	JX205151	[182]
		5S rRNA	76	EF091879	[183]
LAMP		16S rRNA	variable	DQ078785	[185]
			variable	DQ486027	[186]
		*ptp3*	variable	XM_024473556	[148]
*Nosemaapis* and *N. ceranae*					
PCR		ITS region	118–122	AY741110	[213]
multiplex PCR		16S rRNA	218–219	DQ486027	[170]
			321	DQ329034 U26533 DQ078785 DQ286728	[216]
	*N. apis*	16S rRNA	321	DQ329034	[171]
	*N. ceranae*		218	DQ486027	
	*A. mellifera*	RPS5 gene	115	XM_006570237	
	*N. apis*	16S rRNA	224	U97150	[15]
	*N. ceranae*		143	DQ486027	
	*N. apis*	*rpb1*	297	DQ996230	[14]
	*N. ceranae*		662	M_002995356	
*Ascosphaera apis*					
PCR		5.8S rRNA	136	U68313 U18362	[195]
		ITS1-5.8S rRNA-ITS2	525,439	GQ867766	[194]
			486		[193]
rep-PCR		ERIC, BOX, or REP elements	variable	n.a.	[196]
*Aspergillus*					
LAMP	*A. flavus*	18S rRNA	variable	D63696	[210]
PCR	*Aspergillus* spp.	ITS region	variable	n.k.	[98]
		β-tubulin	variable	n.k.	[209]

n.a., not applicable; n.k., not known.

### 2.3. Protozoa

#### 2.3.1. Overview

Protozoa are the least-studied group of bee pathogens (Figure 2). So far, four species of protozoa infecting *A. mellifera* have been identified and are of interest: *Crithidia mellificae* and *Lotmaria passim* of the family Trypanosomatidae, *Apicystis bombi* of Lipotrophidae and *Malpighamoeba mellificae* of Amoebidae. Of the trypanosomatids, *C. mellificae* and *L. passim* both seem to be associated with honeybee health. However, only the latter has shown to have a wide distribution and has been reported to correlate with *Nosema* spp. infections. *A. bombi* is an emerging parasite in *A. mellifera*, established as a bumble-bee pathogen; little is known about its impact on the honeybee. On the other hand, *M. mellificae* is a long-known protozoan species, though it is the least studied in terms of both life cycle and genomics. In general, the interest in protozoa seems to be regionally confined and much remains unknown about their pathogenicity and relevance as hive pathogens.

#### 2.3.2. *Crithidia mellificae* and *Lotmaria passim*

Trypanosomatids are flagellated protozoa of the Kinetoplastida class with at least one invertebrate host during their life cycle [217]. Four species have been found infecting the gut of honeybees: *Crithidia acanthocephali*, *C. bombi*, *C. mellificae* and *Lotmaria passim*, but their relevance to bee health is uncertain [218]. The most well-known of these species are *C. mellificae* and *L. passim*, and the latter has been recognized as a separate species only since 2015 [13,219,220]. *L. passim* is morphologically relatively similar to *C. mellificae*, and upon its description, based on electron microscopy techniques and gene sequencing, it is now believed to be the most prevalent trypanosomatid among honeybees [13,89,221,222]. A seasonal component for *L. passim* beehive infections has been recorded, as well as a positive correlation between *L. passim* and *N. ceranae* loads [89,164]. Infection of bees with these trypanosomatids is associated with an increased lethality and reduction in lifespan. However, although infections take place in the bee gut lumen, no damages to the gut epithelium have been described [223].

In addition to the differentiation of *C. mellificae* and *L. passim* by electron microscopy, selective growth in different media has also been reported and can be useful in future studies on their relevance to bee health [223]. Simple and efficient detection and discrimination between these species can be achieved by molecular methods, such as PCRs based on the 18S rRNA, *rpb1*, GAPDH, and *cytb* genes, one of which has been successfully applied to honey samples (Table 3) [224,225,226,227]. However, PCR assays for the detection of *C. mellificae* developed before the description of *L. passim* need to be reevaluated, because of potential cross-reactivity.

Quantitative PCRs have been developed and applied to epidemiological studies in which the load of *C. mellificae* and *L. passim* is analyzed [162,164]. Additionally, in this case, there is uncertainty regarding the specificity of those methods developed before the separation of these two species. Indeed, the primers reported by Runckel et al. [162] are not *C. mellificae*-specific but also hybridize with *L. passim* DNA (Lucas Lannutti, personal observation).

Duplex PCR and triplex qPCR protocols allow for the simultaneous detection of *C. mellificae* and *L. passim*, and for the latter, specific primers for honeybee DNA are used as internal control [226,228]. The genomes of both pathogens are publicly available, which allows for the future development of improved novel detection and genotyping tools (GenBank Assembly accession no GCA_000635995, [229]; and accession no MDUD00000000, unpublished).

#### 2.3.3. *Apicystis bombi*

A single species of apicomplexan protozoa, *Apicystis bombi,* belonging to the Lipotrophidae family, has been described in *A. mellifera*. The main host of this parasite appears to be the bumble bee, where it has been described infecting fat-body cells resulting in hypertrophism of the fat body [230,231]. This parasite has so far only been found in honeybees in Japan and Argentina, and studies are scarce [232,233]. The relevance of this pathogen for honeybee health, its host specificity and geographic distribution are not well-known and need further study.

The genome of *A. bombi* has not been sequenced yet, and only 18S rRNA gene sequences are available in GenBank [233,234]. Consequently, PCRs exclusively using this gene as target have been developed and applied to the detection of this pathogen in honey and/or bumble bees [231,232,235,236].

#### 2.3.4. *Malpighamoeba mellificae*

*Malpighamoeba mellificae* is a cyst-forming amoeba commonly found to infect the Malpighian tubules of bees, damaging their epithelial cells. Thus, infected bees have impaired excreting capacity and an increased vulnerability to environmental toxins as well as loss of water and ions [237,238]. Amebiasis is transmitted when a bee ingests *M. mellificae* cysts that have been eliminated with the feces of an infected bee. The relatively few studies carried out report that the infection is associated with diarrhea, abdomen swelling, lethargy, and death [238,239,240]. It has been reported that the presence of *M. mellificae* in honeybees correlates with *Nosema* spp. infections [241,242]. Until now, diagnostics has been carried out by microscopic observation of Malpighian tubes [240]. Recently, however, a qPCR diagnostic assay with high specificity and sensitivity has been developed based on the 18S rRNA gene sequence as a target [243]. This will facilitate studying the relevance of this long-known, yet neglected, honeybee pathogen.

**Table 3:** Molecular assays for the detection of protozoan pathogens of the honeybee.

Type of Reaction	Species	Target	Size of Amplicon (nt)	Accession Number	Ref.
*Lotmaria passim*					
PCR		*cytb*	247	KJ684960	[225]
		18S rRNA	459	KM066228 KJ713376 KJ71337	[224]
		GAPDH	402	M066224 KJ713349 KJ71335	
		18S rRNA	163	KM066244	[227]
qPCR		*cytb*	146	KJ684960	[164]
*Crithidia mellificae*					
PCR		*cytb*	140	KJ684951	[225]
		GAPDH	140	KJ713345	[227]
*Lotmaria* and *Crithidia*					
qPCR ^a^	*C. mellificae* (and *L. passim*)	18S rRNA	123/125	KX953204/MN879795	[162] ^a^
multiplex PCR	*L. passim*	*rpb1*	254	LT976800-2	[226]
	*C. mellificae*	GAPDH	177		
	*C. bombi*	TOPII	133		
multiplex qPCR	*L. passim*	*cytb*	184	MG494247 KJ684969	[228]
	*C. mellificae*		146		
*Apicystis bombi*					
PCR		18S rRNA	850	FN546182	[236]
*Malpighamoeba mellificae*					
qPCR		18S rRNA	137	OL757386	[243]

^a^ Molecular assay for *C. mellificae* detection described previous to *L. passim* description by Schwartz et al. [13]. As bioinformatic analysis predicts cross-reaction with *L. passim*, this finding needs experimental confirmation.

### 2.4. Bacteria

#### 2.4.1. Overview

There are relatively few bacterial pathogens that affect honeybees. *Paenibacillus larvae* and *Melissococcus plutonius* are the most prominent and well-characterized bacterial pathogens and have been detected exclusively in honeybees. Other relevant but somewhat neglected and possibly underestimated bacterial pathogens are *Spiroplasma melliferum* and *S. apis*, known to infect honeybees as well as bumble bees. Furthermore, *Serratia marcescens* has been shown to be highly pathogenic, and has been isolated from more than 70 insect species, but also from plants, small mammals and as an opportunistic pathogen, from hospitalized human patients.

#### 2.4.2. *Paenibacillus larvae*, the Causative Agent of American Foulbrood

*Paenibacillus larvae*, a worldwide-distributed, Gram-positive, spore-forming bacterium of the Firmicutes phylum, is the causative agent of American Foulbrood (AFB), one of the most contagious and destructive infections in honeybees [244]. Feeding of larvae with spore-contaminated food by nurse bees allows spores to enter the intestinal lumen of bee larvae, where they develop to the vegetative bacterial stage and invade and multiply in the hemocoel, leading to larval death if infection occurs in the first hours after hatching [245,246,247]. Millions of spores are formed in each infected larva, and as few as eight spores are required to initiate a new infection [248]. Pathogen transmission to naïve larvae occurs through nurse bees or directly by spores that remain at the base of a brood cell. In addition, the infection may pass to other colonies when combs are exchanged or through contaminated honey or transfer of infected queens [83]. Spores are highly resistant to heat and chemical agents and can survive for years. In addition, antibiotics can kill vegetative bacteria but are ineffective against spores. Therefore, destruction of contaminated hives and equipment by fire is one of the few options available to limit dissemination of AFB [249]. Because of its socioeconomic importance and impact on international trade, AFB is a notifiable infection, and one of the six bee diseases listed in the manual of the OIE Manual of Terrestrial Animals [250].

Severe cases of AFB are characterized by a mottled appearance of the combs, resulting from a pattern of healthy capped brood, uncapped cells containing the remains of diseased larvae, and empty cells. These clinical signs have been estimated to appear at a load of 3000 spores per bee [251]. Suspicion of the disease is usually confirmed by a matchstick test, in which the remains of dead larvae are pierced and examined to see if there is a brown, sticky, ropy thread at the tip of the stick. However, false-negative results are obtained in cases where the larval remains are watery [83,250,252]. Several detection methods are suitable for early detection of AFB before onset of clinical signs, but they also allow for confirming *P. larvae* infection after onset of disease, including colony culturing and characterization, biochemical profiling, antibody-based techniques, and microscopy. These approaches are best suited for laboratories without molecular biology equipment, are well-described elsewhere, and are therefore not further covered in this review [250,252,253]. In contrast and as outlined in the introduction, molecular detection by DNA amplification offers the advantages of high sensitivity and specificity as well as rapid achievement of results. In addition, molecular genotyping allows for studying intraspecies variation and tracing the origin of outbreaks, and is applicable to worldwide epidemiological surveying.

*P. larvae*-specific DNA amplification protocols can be applied to spore/cell suspension samples after cultivation or directly to infected larvae, pollen, honey, and wax or beehive debris [254,255,256,257,258]. Commercially available kits can be used for DNA extraction, but when working with cultured bacterial suspensions, brief heating to 95 °C is sufficient for subsequent species identification by PCR [252]. To improve DNA recovery from honey samples, incubation with lysozyme, EDTA and Triton X-100 has been reported prior to availability of commercial DNA extraction kits [256].

Most described standard PCR assays amplify different *P. larvae*-specific DNA regions of the 16S rRNA gene [256,259,260,261,262]. However, additional target genes have been identified, including those coding for a metalloproteinase, tRNA^cys^ and a hydrolase, among others (Table 4) [256,262]. The search for suitable PCR targets has been greatly facilitated since the sequencing of the *P. larvae* genome in 2006 [197]. In addition to a simple PCR format, increased sensitivity and specificity have been achieved with nested and qPCR protocols [258,263,264,265,266,267]. However, their implementation can be complicated due to the higher risk of amplicon contamination in the first and the high cost of equipment and reagents in the latter. Recently, a novel TaqMan^®^ qPCR reaction was published, allowing for a very sensitive quantification of *P. larvae* spores from honey and hive debris, based on a highly conserved single-copy gene encoding for a metalloproteinase [268].

Molecular epidemiological studies analyzing *P. larvae* genotypes are applied to understand the relationships between outbreaks, analyze the distribution of strains with different pathogenic phenotypes, and evaluate the success of control strategies and management. A genotyping technique which has proven useful for the recognition of bacterial strains is the repetitive-element sequence-based PCR (rep-PCR). It consists of the random amplification of repetitive genetic structures such as BOX elements (short repeated sequences whose function is often unknown), enterobacterial repetitive extragenic palindromic (REP) or enterobacterial repetitive intergenic consensus (ERIC) sequences, followed by electrophoresis for subsequent analysis of band sizes by fingerprinting [252]. Using ERIC primers, the five different *P. larvae* genotypes ERIC I, II, III, IV, and V could be discriminated [249,269,270]. ERIC I and ERIC II have been identified in Europe, Australasia, Asia, and North America, however, they may also be present in other geographic regions that have not yet been studied. Genotypes ERIC III-V do not play a role in field samples but rather exist in culture collections or have been isolated from honey samples. Of note, the origin of genotypes ERIC III-V has not been recorded and is therefore unknown [244,269]. The most frequent ERIC I and II genotypes can be distinguished from each other by a multiplex qPCR assay that targets the virulence gene *plx1* (accession no. KC456421), which encodes for a toxin present in ERIC I, but not in ERIC II strains [271]. Importantly, ERIC I to V are associated with diverse phenotypes, one of which is determined by the different time needed to kill bee larvae in an in vitro assay. Analysis of whole-genome sequences of *P. larvae* ERIC I to V genotypes revealed that the phenotype of the slow-killing ERIC I + II genotypes is associated with a significantly decreased genomic inventory of genes encoding virulent factors compared to fast-killing ERIC III–V genotypes. This observation may explain the much higher prevalence of ERIC I + II genotypes since protraction of larvae killing is known to result in a decreased efficiency of hygienic cleaning of nurse bees [272].

Alternative genotyping methods of higher reproducibility and increased resolution of *P. larvae* genotypes have been reported: two multi-locus sequence typing (MLST) systems, each based on a different set of seven housekeeping genes distinguishing 15 and 21 sequence types (ST), respectively [20,273]. Furthermore, a multiple-locus variable number of tandem repeat analysis (MLVA) system differentiating at least 23 variable number of tandem repeats (VNTR types) has been developed [274]. Interestingly, Morrissey et al. [20] proposed a population differentiation of *P. larvae* within the native geographic distribution of the honeybee host (Europe, Africa, Eastern Asia), but no differentiation when the global population was considered. Furthermore, MLST types determined by Morrisey et al. [20] have been stored online. Further analysis of strains using the same methods allows for the determination of additional strain types, which can be deposited directly online and are therefore immediately accessible [275].

Finally, next-generation genome-sequence-typing methods that have a substantially higher discriminatory power than the before-mentioned traditional MLST and ERIC typing schemes have been developed. Although their high cost and the considerable expertise required still prevent their routine application, their increased discriminatory power allows for investigating and tracing outbreaks and studying the structure of the population, and they can be applied to epidemiological surveillance worldwide. The most commonly used whole-genome sequence (WGS)-based approaches include allele-based analysis by wgMLST (whole-genome MLST) or cgMLST (core-genome MLST). In the former approach, alleles of the query isolate are compared in silico across the whole genome by a gene-to-gene approach to a previously established reference allele database of the species under study. In contrast, in the latter, the comparison is made based on a reduced set of genes known to be present in the large majority of isolates, referred to as the core genome, which usually corresponds to 95 to 99% of coding sequences. Alternatively, an analysis of single-nucleotide polymorphisms of a query isolate genome (wgSNP) across a reference whole-genome database can be carried out [276].

Ad hoc cgMLST typing has been used to successfully trace the source of an AFB outbreak in Sweden, which would not have been possible using traditional MLST or ERIC typing [277]. Importantly, a solid wgMLST online scheme composed of 5738 loci that allows for wgMLST genotyping of *P. larvae* by uploading genome sequences of isolates as query was recently established. This wgMLST scheme allowed for genotyping and determining the global population structure of *P. larvae* with unprecedented discriminatory power [21]. In addition, it has been successfully applied to reveal transmission routes and the origin of recent outbreaks of AFB in Slovenia and other countries [21,278].

#### 2.4.3. *Melissococcus plutonius*, Causing European Foulbrood

*Melissococcus plutonius* causes a disease known as European Foulbrood (EFB), considered fatal and distributed around the world [246]. *M. plutonius* is an anaerobic or microaerophilic Gram-positive, non-spore-forming bacterium of the phylum Firmicutes, family Enterococcacea. First described in 1912 as *Streptococcus pluton*, it was reclassified in 1982 as *M. pluton* [279,280] and later redescribed as *M. plutonius* [281]. Larvae are infected with *M. plutonius* upon ingestion of contaminated food from worker bees, and nutrient deficiencies result from the multiplication of the bacterium in their guts [282]. In addition, virulence and mortality of this bacterial pathogen seem to be associated with the presence of a plasmid encoding the toxin melissotoxin A, which is likely to have detrimental effects on gut cells [283,284].

EFB is listed in the OIE Manual of Terrestrial Animals [283], and although EFB and AFB are caused by different pathogens, due to the similarities of both diseases, EFB is often erroneously referred to as a mild version of AFB. In both cases, larvae are affected, mortality is higher when infection occurs at early stages after hatching, and blotchy patterns are observed in combs. However, AFB and EFB differ in some aspects such as the appearance of predominantly uncapped or capped brood in affected hives, respectively. Additionally, in AFB, larvae die in an upright position and form black scales at the bottom of the cells that are difficult to remove; while in EFB, larvae appear twisted and contorted, and form an easily removable, rubbery brown scale. Importantly, although *M. plutonius* does not form spores like *P. larvae*, contaminated material remains infective for many years [83,280,282].

Because of the difficulties associated with in vitro cultivation of *M. plutonius* and the fact that cultured strains are nonvirulent unless coinfection with other bacteria occurs, molecular diagnosis is preferred over cultivation for early diagnosis of EFB [282,285,286]. The determination of the complete sequence of the *M. plutonius* genome, available since 2011, has accelerated the development of molecular diagnostics and allowed for a deeper understanding of strain differences and molecular epidemiology of this bacterium [283,287,288,289].

Similar to *P. larvae*, *M. plutonius* genomic DNA can be extracted from samples of adult and larvae bee stages or pollen and hive debris, and the 16S rRNA gene has mainly been used as the target gene for molecular detection by direct, seminested or qPCR (Table 4) [290]. Other gene targets were also used, including the melissotoxin A plasmid-encoded gene for detection by standard PCR, and the *sodA* (manganese-dependent superoxide dismutase) gene for detection by qPCR [284,291].

In addition to the above-mentioned PCR-based diagnostics, two alternative *M. plutonius* DNA detection protocols are particularly useful for the screening of large numbers of samples: (i) a LAMP protocol targeting the *gyrB* (DNA gyrase subunit B) gene, and (ii) a colorimetric nanoparticle-based detection method. The first method has the advantages of showing a 10 times improved detection limit compared to a seminested PCR based on the 16S rRNA gene, and it does not require special equipment to analyze the results, while the second is characterized by its speed and low cost [292,293,294].

MLST based on four highly polymorphic genes allows for distinguishing altogether 27 different STs, clustering into the three different clonal complexes, CC12, CC3 and CC13 (Table 4) [19,295,296]. *M. plutonius* strains pertaining to CC12 comprise “atypical strains” with less demanding cultivation requirements than those of STs of “typical strains” that pertain to CC3 and CC13. In contrast, strains of CC12 display a higher virulence than those of CC3, while strains of CC13 are avirulent [297,298]. A duplex PCR with a good detection performance targeting the *napA* (Na^+^/H^+^ antiporter) gene for identification of typical strains (CC3 and CC13) and the *Fur* (ferric uptake-regulator family) gene, for the detection of atypical strains (CC12), is available (Table 4) [297,299]. Importantly, later studies could not find a relationship between MLST STs and/or clonal complexes and virulence; an increased virulence was rather associated with the presence of a plasmid encoding melissotoxin A and other virulent factors [284].

Of note, there are multiplex PCR and multiplex qPCR assays that allow for simultaneous detection of *P. larvae* and *M. plutonius*, one of which also detects the presence of the fungal bee pathogen *A. apis* [195,300].

#### 2.4.4. *Spiroplasma apis* and *S. melliferum* Causatives Agents of May Disease

*Spiroplasma apis* and *S. melliferum* are Gram-positive bacteria that belong to the phylum Tenericutes, class Mollicutes. Both have a helicoidal shape and no cell wall, are motile, and typically coinfect the hemolymph of bees; however, in many cases, single infections are also observed [301,302]. Details on their pathogenicity mechanisms in honeybees are scarce, but are likely associated with the capacity of these bacteria to pass from the midgut epithelium to the hemocoel, and to infect and multiply intracellularly in tissues and extracellularly in the hemolymph, leading to motility loss and death [247,301]. It has been experimentally demonstrated that infections with *S. apis* are associated with higher mortality rates than infections with *S. melliferum*, though the latter appears to be more geographically widespread [302].

*Spiroplasma* spp. were tentatively identified as the causative agents of May disease, a neurological illness reported for European bees during spring and associated with flowering peaks. Since these bacteria are present in nectar, it is possible that bees are infected through contact with flowers [303,304]. The seasonality of *S. melliferum* infection of bees with a high peak during May has also been reported in the USA [305]. However, another study showed that *S. apis* and *S. melliferum* may not be exclusive springtime pathogens as they could be found in beehives throughout the year in the USA and Brazil, though each species has a unique pattern of prevalence that depends on temporal, geographic and climatic variables, influencing honeybee health and disease cycles [302].

Molecular studies include the sequencing of the genomes of these pathogens and the development of molecular detection methods (Table 4) [306,307]. A multiplex PCR has been designed based on species-specific primers to amplify the *S. melliferum spiralin* and the *S. apis rpoB* genes, which were combined with universal primers against *Spiroplasma* sp., allowing for detecting both species after isolation of each bacteria [308]. In contrast, a qPCR assay using *S. melliferum spiralin* primers of Meeus et al. [308] and *S. apis* 16S rRNA primers, allowed for highly sensitive species-specific detection [302].

#### 2.4.5. *Serratia marcescens* Causing Honeybee Sepsis

*Serratia marcescens* is a ubiquitous Gram-negative bacterium of the phylum Protobacteria, order Enterobacterales, that can cause sepsis in animals and plants [309]. Studies on *S. marcescens*, such as the sequencing of its genome and the characterization of different isolates, have been mainly focused on its relevance for human health [310]. The first reports on sepsis in *A. mellifera* are from almost a century ago, but important advances in the understanding of the pathogenicity of *S. marcescens* in this insect took place only recently [311,312]. Studies suggest that this bacterium is frequently found in the bee microbiota and acts as an opportunistic pathogen that turns lethal when conditions are adequate [313,314].

A few reports on molecular diagnosis of *S. marcescens* in bees are based on sequenced regions of the 16S rRNA gene amplified by PCR using universal primers, and are not adequate for species-specific detection [314,315]. For this reason, development of molecular diagnostics for specific and rapid detection as well as genotyping would facilitate the research on this pathogen. Importantly, 16S rRNA amplicon analysis by denaturing-gradient gel electrophoresis (DGGE) allowed for the identification of a putative novel *S. marcescens* strain [315]. Of note, *Spiroplasma* spp. and *S. marcescens* have been frequently found with high prevalence in studies of the bee microbiome and of bee pests such as *V. destructor* and AFB, an observation that corroborates that both of these species are opportunistic pathogens [316,317].

**Table 4:** Molecular assays for the detection, interspecific differentiation and intraspecific genotyping of bacterial pathogens of the honeybee.

Type of Reaction	Species, Strains or Genotype	Target	Amplicon Size (nt), Sequence Types and Profiles	Accession Number and Amplification Primers	Ref.
*Paenibacillus larvae*					
PCR		16S rRNA	973	X60619	[259]
			1106	AY030079	[260]
			700		[261]
			237,374,451,695,739		[256]
			665,965		[262]
		metalloproteinase	155,242,342	AF111421	[256]
			273		[262]
	ERIC I + II	ERIC amplicon of 970bp	550	n.a.	[318]
nPCR		16S rRNA	572 (final amplicon)	AY030079	[263]
qPCR		16S rRNA	233	U85263	[264]
			74	X60619	[265]
			380	AY030079	[266]
			130	CP019687, locus tag BXP28_01730	[258]
			167		[267]
multiplex qPCR	ERIC I + II	16S rRNA	249	AY530294	[271,319]
	ERIC I + II	*ItuC*	209	CP019717	
	ERIC I	*plx1*	176	KC456421	
rep-PCR	ERIC I-V genotype	ERIC sequences	ERIC profiles I-V	ERIC1R-ERIC2 primer pair	[320]
MLST	ST (sequence type) 1-21 + 24,25	*clpC*, *ftsA*, *glpF*, *glpT*, *Natrans*, *rpoB*, *sigF*	ST1–21 ST1-7, 13-21: ERIC I; ST10-12,24: ERIC II; ST8-9: ERIC III/IV; ST25:ERIC V	HG530076-109 of target genes and alleles	[20]
	ST 1-15	*ilvD*, *tri*, *purH*, *recF*, *pyre*, *SucC*, *glpF*	ST1–15 ST1-2,7-15: ERIC I; ST4: ERIC II; ST6: ERIC III, ST3,5: ERIC IV	KY673263-528 of target genes and alleles	[273]
MLVA	MLVA type 1-23	VNTRs	MLVA1-23; MLVA1-17: ERIC I MLVA18-21: ERIC II MLVA22: ERICIII; MLVA23: ERIV IV	VNTR primer pairs A-E	[274]
solid wgMLST	wgMLST types	reference whole genome including 5745 loci derived from comparison of 179 genomes	much improved discrimination compared to traditional MLST	n.a.	[21]
*Melissococcus plutonius*					
PCR		16S rRNA	812	X75752	[321]
		melissotoxin A	1360	KMT29105	[284]
multiplex PCR	typical strains (CC3, CC13)	*napA*	187	AB778538	[299]
	atypical strains (C12)	*Fur*	424	BAL62104	
seminested PCR		16S rRNA	486,276	X757551	[292,322]
qPCR		*sodA*	79	EF666055	[291]
		16S rRNA	69	AJ301842	[323]
LAMP		*gyrB*	variable	AP012200, locus_tag MPTP_0005	[294]
MLST	STs cluster into clonal complexes CC3, 13 and CC12	*argE*, *galK*, *gbpB*, *purR*	ST1-27; ST1, 4,8,9,13,14, 15,17,18,20,26: CC13; ST2,3,4,5,6,7,11,12,22,23,24: CC3 ST10,12,16,19,21,25, 27: CC12	HF569117-42	[19,295] [296] [297]
Nanoparticle-based detection		cell wall-associated protease gene	n.a.	NC_015516	[293]
*P. larvae* and *M. plutonius*					
multiplex qPCR	*P. larvae*	*tnp60*	87	CP003355	[300]
	*M. plutonius*	*napA*	92	AB778538	
	^a^ *A. mellifera*	*actin*	87	AB023025	
multiplex PCR	*P. larvae*	16S rRNA	973		[195,259]
	*M. plutonius*		281	*M. plutonius*: ^b^ msa based on AY862507, AJ301842, X75751-2	
	^c^ *Ascosphaera apis*	5.8S rRNA	136	*A. apis*: ^b^ msa^.^ based on U68313, U18362	
*Spiroplasma apis* and *S. melliferum*					
multiplex PCR	*S. apis* and *S. melliferum*	*1*6S rRNA	976	JN628939	[308]
	*S. apis*	*rpoB*	636	DQ313816	
	*S. melliferum*	*spiralin*	160	M59366	
multiplex qPCR	*S. apis*	16S rRNA-ITS1	190	AY736030	[302]
	*S. melliferum*	*spiralin*	160	M59366	
	*A. mellifera*	RPS5 gene	115	GB11132	

n.a., not applicable; ^a^ primers binding to the actin gene of the honeybee are used in this molecular assay as control to verify quality of template DNA; ^b^ msa, multiple sequence alignment; ^c^ *Ascosphaera apis*, fungi causing chalkbrood disease of the honeybee larvae.

### 2.5. Virus

#### 2.5.1. Overview

Among the pathogens that affect honeybee health, viruses are a dynamic group with an increasing number of members. Bee viruses cause significant losses in honey production around the world and are associated with high morbidity and mortality in both naïve and wild bees [5,30,324]. Although viruses are usually present in colonies without causing clinical signs, under stress conditions, immunosuppression or nutritional deficiencies, viral infections may produce associated diseases and weaken colonies. Furthermore, bee viruses are transmitted by various routes and easily spread within the colony.

The emergence of *V. destructor* as a vector of viruses in *A. mellifera* facilitated the spread of viral diseases, triggering new threats to honeybee health (see Section 2.1.2). Indeed, numerous viral disease outbreaks including acute bee paralysis virus (ABPV), chronic bee paralysis virus (CBPV), slow paralysis virus (SPV), black queen cell virus (BQCV), Kakugo virus (KV), cloudy wing virus (CWV), sacbrood bee virus (SBV), and deformed-wing virus (DWV) have been documented to be linked to the presence of *V. destructor*, which acts as a mechanical and/or biological vector [240,325,326,327,328].

Most bee viruses belong to the families *Districtoviridae* (Israeli acute bee paralysis virus, IAPV; Kashmer bee virus, KBV; acute bee paralysis virus, ABPV; BQCV) and *Iflaviridae* (DWV, KV, *Varroa destructor* virus 1, VDV-1/DWV-B, SBV; slow bee paralysis virus, SBPV), while some are unclassified (CBPV; Lake Sinaí virus, LSVs). Recent advances in metagenomics have allowed us to identify new viruses in bees and other pollinators; including bee macula-like virus (BeeMLV) from the *Tymoviridae* family, *Osmia cornuta* nudivirus (OcNV) from the *Nudaviridae* family, Apis rhabdovirus (ARV-1), *A. mellifera* iflavirus, and *A. mellifera* feranovirus, among others [329,330].

Most genomes of bee viruses are composed of positive-sense single-strand RNA (+ssRNA). RNA viruses have high mutation rates and often form diverse populations of variants or quasi-species [331]. In contrast, very few viruses with DNA genomes have been identified in bees, such as *A. mellifera* filamentous virus (AmFV) [330] and OcNV [332].

Since bee viruses do not replicate efficiently in cell culture, the effect of viral replication in the host cell cannot be assessed. Thus, infections by bee viruses are classified as overt and covert infections according to the presence or absence of clinical signs, respectively. Covert infection appears when a high replication level of the viral agent is reached in affected tissues or organs, and favors horizontal transmission. In overt infections, on the other hand, vertical transmission is the preferred route of virus spread, and the infection typically persists in the population for many generations. The latter is the most common way viruses infect colonies, but as stated before, under adverse conditions, overt infections may turn into covert infections. In addition, different viral pathogens can infect colonies simultaneously and interact with each other, increasing their pathogenic effect. Diagnosis of bee viruses by conventional methodologies is complicated due to the lack of appropriate cultures, common clinical signs of different viral infections, and the high frequency of multiple infections in the individual bee and/or colony. Thus, detection of bee viruses is mainly carried out by molecular diagnostics, focusing on the amplification of a particular specific sequence region of the viral genome. Nucleic acid amplification by conventional RT-PCR has been widely used for the detection of many bee viruses [333]. Molecular diagnostics based on PCR has been demonstrated to be a powerful tool to detect bee viruses with high sensitivity and specificity; however, the previously required nucleic acid extraction is tedious and time-consuming. In an attempt to overcome this limitation, Huang et al. [334] developed a novel, simpler method for detecting viral infections in honeybees based on the analysis of a very small volume of hemolymph by direct RT-PCR. Several bee viruses, including DWV, BQCV and SBV, were reliably detected by this method up to a dilution of 1 in 1000 of hemolymph, yet the exact detection limit of the virus particle titer was not reported by the authors.

Of note, LAMP has been demonstrated to be a rapid, cost-effective, and robust technology that can be used with crude extracts, avoiding time-consuming purification of nucleic acids. For detection of bee viruses, LAMP assays have only been developed for country-specific SBV outbreaks. The developed LAMP assays target the polymerase or viral protein gene of SBV with a similar analytical sensitivity to RT-qPCRs (reviewed in [17]).

In recent years, RT-qPCR assays have become the preferred technique for bee virus detection due to their high sensitivity and the possibility of multiplexing, quantification and high-throughput processing [335,336]. More recently, metagenomics and next-generation sequencing have allowed for discovering many new viral agents, not only in bees, but also in other social insects.

#### 2.5.2. Molecular Detection of Common Bee Viruses and Their Variants

The most commonly detected viruses associated with economic losses worldwide are ABPV, BQCV, CBPV, DWV, KBV, IAPV and SBV. Studies describing the presence, abundance or prevalence of these viruses utilize either end-point RT-PCR or RT-qPCR assays, and there is an enormous number of specific primers available designed for this purpose. In the context of this review, we will focus on newly developed molecular approaches and on recently reported field studies applying previously developed assays. In Table 5, the corresponding molecular approaches and assays are summarized.

The deformed-wing virus is one of the main viruses affecting the honeybee. As mentioned above, DWV belongs to the genus *Iflavirus*, (+)ssRNA. Three variants of DWV have been described, comprising DWV-A, DWV-B (also known as *V. destructor* virus-1), and DWV-C. DWV-C variants group all the sequences resulting from recombination between DWV-A and DWV-B [337,338,339,340,341].

DWV spreads via different transmission routes and *V. destructor* functions as a highly efficient vector within the honeybee colony [338,342]. This virus infects all bee castes (drone, worker, queen) and has been detected in all developmental stages of *A. mellifera* (egg, larvae, pupae, adult). Virus infection is associated with the appearance of bees with deformities in the wings and results in a shortened life expectancy, manifested at the colony level by a progressive decrease in the size of the population [343,344].

The most common method for DWV detection is RT-PCR. A variety of primers have been described that amplify different regions of the virus genome encoding structural and non-structural proteins. Most of the amplification primers target the RNA-dependent RNA polymerase gene (RdRp) [331,340,342,345]. Others target regions coding for the internal ribosome entry site (IRES), helicase, and structural proteins such as VP1 and VP2 [331,337,340,341,342,346,347,348,349].

Recently, a real-time, micro-scale, chip-based PCR system allowing for rapid detection of viral RNA was described. This methodology amplifies a RdRp region through the use of DNA chips, a technique that identifies a specific nucleotide sequence through DNA hybridization [350]. On the other hand, multiplex PCR has been developed for the detection of several viruses that affect bees, including DWV [351,352], using the above-mentioned genes as targets. Thus, adequate and reliable tools for the detection of any DWV variants and coinfecting viruses are available for research groups.

The advancements in sequencing methodologies have provided the possibility of implementing the analysis of complete DWV genomes in such a way that recombination points between the DWV-A and DWV-B variants can be determined, circulating strains originating in different regions can be characterized, and molecular differences between covertly circulating DWV strains or highly virulent viruses can be identified [340,346,349,353,354,355].

Another virus closely related to DWV is the Kakugo virus (KV), with which it shares 96% nucleotide identity. Both are often considered to be variants of a single species complex [346,356]. As with DWV, the most applied methodology for KV detection is RT-PCR or RT-qPCR, targeting regions that encode the nonstructural protein RdRp or structural proteins such as VP1 [345,357,358,359,360].

Sacbrood virus (SBV) also belongs to *Iflavirus*; its detection by molecular assays such as RT-PCR or RT-qPCR uses specific regions encoding capsid proteins or non-structural proteins such as helicase and RdRp [345,360,361,362,363]. Widely used multiplex PCRs have been developed for the detection of SBV together with other viruses that coinfect *A. mellifera* colonies [347,348,351,363,364].

Moku virus (MV) is an *Iflavirus* first discovered in the predatory social wasp. In a recent study testing for the presence of MV by RT-qPCR, 43% of honeybee colonies (*n* = 69) resulted positive; MV replication could be confirmed in *A. mellifera* and *Vespidae* species [365].

Lake Sinai viruses (LSV) are (+)ssRNA viruses (*Sinaivirus*) that affect honeybees and were recently detected through metagenomics studies [162]. Infections by LSV can reach high levels, and are not associated with overt disease but modulate the infection dynamics of other pathogens [366]. LSVs have a cosmopolitan distribution and are among the most abundant of the above-described groups. However, the epidemiological factors determining the distribution of virus variants in honeybees are still unknown [367]. Although the genome of LSV has been detected in *V. destructor* [368], LSV abundance and *Varroa* infestation levels are not associated, suggesting that this mite is not a vector of LSV [366].

Since conventional diagnosis by clinical signs is not possible, molecular assays are indispensable for the detection of LSV. Even so, the high genetic diversity of LSV haplotypes hampers an exact assessment of LSV prevalence by PCR. Recently, Iwanowicz et al. [369] reported a PCR assay based on minimal degenerated amplification primers targeting the polymerase *orf*, which was capable to detect all known variants of LSVs. Furthermore, the generated 365 bp amplicon was suitable for metagenomic sequencing and allowed for the study of the association of genetic variation with host range, virulence, transmission, and other features of the epidemiology of *Sinaivirus*.

Chronic bee paralysis virus (CBPV) is a bipartite, (+)ss RNA virus. Although it was the first bee virus isolated and described from honeybees [24], its family and genus are currently unassigned. CBPV has a worldwide distribution and it usually persists as covert infection in honeybee colonies; however, overt infections have been reported and confirmed by RT-PCR. The virus affects colonies with two different types of clinical signs. The first type includes paralysis, trembling and crawling of bees, and the second includes hairless, black bees, with shortened abdomens. In both cases, the colony suffers massive losses of worker bees [240].

Detection and quantification of CBPV based on RT-qPCR assays have been reported. Blanchard et al. [370] developed a two-step RT-qPCR assay that targets the RdRP-encoding gene and found a correlation between a high CBPV genomic load and pathological signs when testing it on symptomatic and dead bees. Importantly, this assay was further validated according to the ISO/IEC 17025 and the XP U47-600 standard issued by the French Standards Institute [371]. Quantification of CBPV viral loads in *V. destructor* could be determined by Celle et al. [372]; however, this mite is rather considered a natural reservoir of CBPV than a vector.

The acute bee paralysis virus (ABPV), Kashmir bee virus (KBV), and Israeli acute paralysis virus (IAPV), form the ABPV-KBV-IAPV complex. Infection by each of the three viruses shows a similar pathology at the level of the individual bee and the colony. Like most members of *Dicistroviridae* (+ssRNA), these viruses normally persist as covert infections within the colony, and are detectable by RT-PCR assays [45].

Since ABPV, KBV and IAPV are genetically very similar, there is a potential risk of misdiagnosis including false-positive and false-negative detection, and strain misassignment [45]. To avoid this, accurate diagnosis can be achieved by RT-PCR followed by sequencing, with the added benefit of obtaining phylogenetic information on these viruses [373,374,375]. Several RT-qPCR assays targeting structural proteins or RdRP-encoding genes have been reported for the detection of the ABPV-KBV-IAPV complex [45,374,376,377,378,379].

IAPV is one of the most studied viruses because it can infect every member of the colony and its detection strongly correlates with colony collapse disorder (CCD) [380,381,382]. IAPV can persist as an overt infection, but under certain conditions that favor the presence of high quantities of virus, covert infections and typical clinical signs of IAPV appear [376,383,384]. As for other honeybee viruses, *V. destructor* is an effective vector of IAPV [385].

IAPV covert infection induces a broad spectrum of clinical signs including paralysis and body trembling. However, whether the neuron system is the preferred tropism of the virus has not yet been resolved [386]. In a study conducted to investigate viral abundance in different tissues of honeybees, the tracheae showed a greater viral load than other tissues. In addition, large quantities of virus in tracheae induced a notorious downregulation of succinate dehydrogenase and cytochrome c oxidase genes. The authors concluded that paralytic signs or trembling mitigated tachypnea induced by IAPV infection [386].

IAPV is a positive single-stranded RNA virus that was first described as a distinct pathogen from KBV and ABPV based on its genome sequence in Israel in 2004 [381]. IAPV belongs to the family *Dicistroviridae* and has two ORFs each encoding for a polyprotein, and each preceded by an internal ribosomal entry site (IRES) [374]. In a phylogenetic analysis of IAPV, Palacios et al. [374] reported the existence of at least three distinct IAPV lineages; the authors also suggested the possibility of recombination events and identified differences in coding sequences that may have implications for virulence.

Several RT-PCRs assays have been developed to detect IAPV [374,380,381,383]; however, detection of IAPV is complicated due to the high level of sequence variation in the viral genome [384] and a high degree of conservation with related viruses. Thus, sequence analysis has been recommended for IAPV identification [374].

Tai Truong et al. [387] developed a detection methodology based on amplification of multiple sites within the IAPV genome and ultra-rapid, real-time polymerase chain reaction (UR-qPCR) combined with a freezing–thawing method for RNA isolation and micro-scale, chip-based PCR. Primers target the RNA-dependent RNA polymerase gene (RdRp) and two capsid genes (VP3 and VP1) in order to increase the detection of IAPV variants.

One of the most prevalent honeybee viruses is the black queen cell virus, which affects developing queen larvae and pupae during the capped-cell stage. BQCV belongs to the *Districtoviridae* family and has a similar genome structure to ABPV and KBV. Transmission is driven mainly by nurse honeybees that horizontally transfer BQCV from infected cells to healthy larvae in brood food, but queens may also vertically transmit it to eggs. It has been shown that BQCV is associated with the infection of the microsporidian *N. apis* [47]. This virus has been found in high abundance in collapsing colonies and is commonly detected by RT-PCR together with the ABPV-KBV-IAPV complex and DWV. *Orf*2 and the 5′UTR region are most frequently chosen as targets for nucleic acid amplification [162,388,389,390].

#### 2.5.3. Application of Molecular Diagnostics for the Detection of Multiple Viruses

Most studies on bee viruses analyze the presence of several, including DWV, BQCV, ABPV, CBPV, IAPV and SBV. In early developments, Benjeddou et al. [390] applied RT-PCRs to detect ABPV and BQCV based on the amplification of a region of the 5′ end of the genome.

Moharrami and Modirrousta [391] described the presence of the above-mentioned six viruses after sampling 23 provinces of Iran by end-point RT-PCR [388]. The authors found that BQCV was the most prevalent, followed by DWV. In a multiyear survey in the US, honeybee diseases were evaluated by measuring the incidence and levels of *V. destructor*, *Nosema* spp., and a diverse set of honeybee viruses. All viral agents were detected by RT-qPCR using either previously reported primers sets [162,389,392] or primers developed for the detection of CBPV and SBPV by the authors [393].

In a prospective study, Runcket et al. [162] investigated seasonal patterns of pathogens in a migratory bee-keeping operation. Virus detection was carried out by microarrays, end-point PCR, qPCR, and ultra-deep sequencing, and several sets of primers targeting DNA of different viruses were developed in this study. Results showed that the peak infection of common honeybee viruses occurred in the summer, with the exception of Lake Sinai virus 2 (LSV2) which peaked in January. Another study explored seasonal patterns of pathogen presence and abundance, and the impact of viruses on honeybee colony health, over one year in commercially managed colonies involved in almond pollination [394]. LSVs together with BQCV, DWV and SBV were the most frequently detected viruses by RT-PCR [162,368]. Results obtained in this study suggested an inverse relationship between LSV2 abundance and colony health.

Similar results were reported by Cavigli et al. [395], who showed that pathogen prevalence and abundance were associated with both sampling date and beekeeping operation, and that weak colonies had a significantly higher mean pathogen prevalence than strong colonies. In this study, the authors used various previously reported RT-PCR and RT-qPCR assays [162,324,368,396,397,398].

In order to identify synergistic interactions between pathogens and/or parasites that may be involved in colony death, D’Alvise et al. [399] studied the presence of diverse viruses, as well as bacteria and parasites in individual bees by RT-qPCR using sets of primers described by Locke [389] and Papp et al. [400]. BQCV, LSV and DWV-B (VDV1) were found to be the most abundant viruses, followed by ABPV, CBPV, SBV, DWV-A, Aphid lethal paralysis virus (ALPV), IAPV, iridescent invertebrate virus (IIV) and KBV.

Molineri et al. [360] compared the presence of honeybee viruses in different climatic regions from Argentina. They examined 385 colonies distributed in five Argentine ecoregions to study the presence of seven different virus species with infestation by *V. destructor.* Detection of all viruses was performed by RT-qPCR using primers developed by Locke et al. [389]. Five viruses were detected in colonies with the following prevalence: DWV (35%), ABPV (21.5%), BQCV (8.0%), CBPV (2.2%), and SBV (1.1%), while KBV and IAPV were not detected. Furthermore, DWV and ABPV were found to be associated with infestation of *V. destructor*.

Several multiplex PCR assays have been designed for honeybee viruses [22,23,401,402,403]. Sguazza et al. [23] developed a multiplex PCR for rapid and simultaneous detection of altogether six bee viruses. The latter assay allows the rapid and cost-effective detection of the most prevalent honeybee viruses, and became a useful tool to study the dynamics of viral infections and in surveillance studies.

Real-time qPCR can also be multiplexed, usually for the simultaneous amplification of a target and internal reference standards, by using TaqMan^®^ probes with different fluorophores. The main reason for multiplexing is to save cost and time; however, multiplex qPCR is less sensitive than uniplex qPCR and more complex to optimize. Multiplexing is far more effective through a microarray. Numerous honeybee microarrays have been designed, including honeybee immune gene-pathogen arrays [162,379] and a honeybee virus array [404]. For research purposes, microarrays are currently being replaced by high-throughput sequencing technologies, but retain a future in routine screening applications due to their adaptability and high multiplexing capacity [404].

Real-time PCR assays allow for the quantification of viral load (VL) and determination of thresholds at which clinical signs are observed, so that VL levels can be compared between different seasons or between healthy and diseased bees. Correspondingly, the VLs of six honeybee viruses (ABPV, BQCV, CBPV, DWV, LSV3, and SBV) have been determined and compared in adult honeybees with and without clinical signs. Two newly developed RT-qPCR assays for the detection of LSV3 and SBV were applied in this investigation. Importantly, statistically significant differences between the VL of positive samples were identified between healthy and clinically affected honeybees for ABPV, CBPV, DWV, and SBV, while for BQCV and LSV3, no statistical differences were observed [405]. Moreover, Schurr et al. [406] assessed the accuracy of RT-qPCR (TaqMan^®^) for quantifying ABPV, BQCV, DWV, VDV1 and SBV VLs in naturally infected honeybees with and without clinical signs. After analyzing the VL distribution in both groups, it was possible to define a threshold that allowed for differentiating between covert and overt infections.

The pattern of infection of DWV-A and B variants was analyzed at the intra-colonial level by quantification of bee virus RNA in pupae and larvae using RT-qPCR. To this end, VLs from *V. destructor*-parasitized pupae of known patrilines were determined at different levels of mite infestation. The results showed different patterns of intra-colonial variation for both variants of DWV, suggesting more complex spatiotemporal dynamics of bee virus infections than was previously thought [407].

#### 2.5.4. Recently Identified Bee Viruses by Metagenomics

The development and optimization of high-throughput sequencing technologies make it possible to sequence the genetic material of the entire community of viruses present within a host, without any prior knowledge of viral genome sequences. This approach is known as viral metagenomic analysis. Metagenomics allows for a better characterization of viral diversity and accelerates the identification of novel viruses affecting honeybees and other pollinators [162,332,356,367,408,409,410,411,412]. By this approach, more than 30 additional, previously unrecognized, viruses could be identified [332]. Although they were classified as honeybee viruses, many of them infect a wide variety of insect hosts. This is particularly important under the perspective of the pollinator network, since the presence of multi-host pathogens favors interspecies virus transmission [413].

Recent studies have reported the identification of additional honeybee-infecting (+)ssRNA viruses including the bee macula-like virus (BeeMLV) of the *Tymoviridae* family, *A. mellifera* flavivirus (AFV) and *A. mellifera* nora virus 1 (ANV) [414]. In addition, high-throughput sequencing has enabled the discovery the first negative-sense ssRNA (-)ssRNA viruses which belong to the *Rhabdoviridae*: *A. mellifera* rhabdovirus-1 (ARV-1) [415] and *A. mellifera* rhabdovirus-2 (ARV-2) [416].

Galbraith et al. [332] performed a massive study involving 12 bee species collected from nine countries across five different continents; the authors developed a novel metagenomics pipeline for the rapid and inexpensive screening of bee viruses that included purification of encapsulated RNA/DNA viruses, sequence-independent amplification, high-throughput sequencing, integrated assembly of contigs, and filtering, to identify contigs specifically corresponding to viral sequences. Besides common bee viruses, new viral agents could be identified, including: (i) seco-like virus sharing sequence homology with *Secoviridae*, (order *Picornavirales*); (ii) a novel (+)ssRNA virus within the family *Nodaviridae*, (iii) a tymo-like virus, (iv) *A. mellifera* Rhabdovirus 1 (ARV-1) (family *Rhabdoviridae*), (v) a partiti-like virus (family *Partitiviridae*), and (vi) a circo-like virus (family *Circoviridae*)

Viromes from the Western honeybee subspecies *A. m. ligustica*, *A. m. syriaca*, *A. m. intermissa*, and *A. cerana* and their respective *V. destructor* mites, were analyzed by RNA metagenomics to compare the composition of their viral populations. In this study, two novel viruses: *Varroa* orthomyxovirus-1 (VOV-1) in *A. mellifera* and *V. destructor*, and a Hubei like-virga virus-14 homolog in *V. destructor* were reported, as well as some recently described viruses: ARV-1, BRV-1, VDV-2, and VDV-3. by [417]. It can be expected that novel viruses will be identified in the honeybee by applying metagenomic approaches in the near future.

**Table 5:** Molecular diagnostics for detection and variant differentiation of viruses that infect honeybees.

Type of Reaction	Species or Genotypes	Target	Size of Amplicon (nt)	Accession Number	Ref.
Acute Bee Paralysis Virus (ABPV)					
RT-PCR		VP1	900	AF150629	[390]
		intergenic region, VP2, VP4, VP3, VP3, VP1, VP1	722,788,686,619, 398,858,687		[373]
		RdRP	452		[326]
		VP3	618		[388]
RT-qPCR (SYBR Green)		RdRP	66	AF150629	[418]
			178	NC_002548	[378]
			177	AF150629	[324]
		VP1	197	AF150629	[389]
RT-qPCR (TaqMan)		VP3	67	AF263733	[328]
		ORF2	nk	AF126050	[419]
Aphid lethal paralysis virus strain Brookings (ALP-Br)					
RT-PCR		helicase	464	Q871932	[162]
RT-qPCR (SYBR Green)		helicase	141	Q871932	[162]
Apis iridiscent virus					
RT-qPCR (TaqMan)		major capsid protein	95	AF042340	[328]
Big Sioux River virus (BSRV)					
RT-PCR		protease	519	GF423195	[162]
RT-qPCR (SYBR Green)		5′UTR	281	n.k.	[162]
Black Queen Cell Virus (BQCV)					
RT-PCR		ORF2	700	NC_003784	[390]
		RdRP	424	AF183905	[326]
		5′UTR	472	AF125252	[388]
		capsid/3′UTR	700	NC003784	[420]
RT-qPCR (SYBR Green)		helicase	107	AF125252	[418]
		ORF2	141	NC_003784	[162]
			294		[389]
RT-qPCR (TaqMan)		capsid polyprotein	71	NC003784	[328]
Chronic Bee Paralysis Virus (CBPV)					
RT-PCR		RdRP	445	AF375659	[398]
		5′UTR	315	AF375659	[388]
RT-qPCR (SYBR Green)		RdRP	97	AF375659	[418]
		RdRP	148	EU122229	[394]
RT-qPCR (TaqMan)		RdRP	101	EU122229	[371]
			57	FJ345309	[421]
Deformed-Wing Virus (DWV)					
RT-PCR		structural polyprotein	194	AY292384	[401]
		polyprotein	434	AJ489744	[388]
		helicase	174		[334]
		non-structural proteins	205		[422]
	DWV A	structural and non-structural proteins	variable	AJ489744	[423]
	DWV B		variable	NC_006494	
	DWV A	capsid	424	NC004830	[420]
	DWV B		528		
	DWV C		446		
RT-qPCR (SYBR Green)	DWV A	3Cpro	136	n.k.	[389]
	DWV B	L	413	n.k.	
	DWV A and DWV B	helicase	179	AY292384	[341]
	DWV A	VP2	211	AY292384	
	DWV B	IRES (internal ribosome entry site)	116	AY251269	
			69		[418]
	DWV A	RdRP	155	NC_004830	[331]
	DWV B		155	AY_251269	
	DWV C		152	CEND01000001	
	DWV A	IRES	118	AJ489744	[423]
	DWV B		117	NC_006494	
	DWV A	structural proteins	97	AJ489744	
	DWV B		97	NC_006494	
	DWV A	non-structural proteins	101	AJ489744	
	DWV B		101	NC_006494	
	DWV A	helicase	186	AY292384	[422]
	DWV B		189	AY292384	
RT-qPCR (TaqMan)		helicase	702	NC_004830	[396]
		RdRP	114		[328]
		polyprotein	67	HM067437	[421]
	DWV A	VP3	72	AY292384	[406]
	DWV B		73	AY251269	
Israeli Acute Paralysis Virus (IAPV)					
RT-PCR		3′UTR	475	NC_009025	[381]
		capsid	840	NC009025	[420]
RT-PCR		intergenic region, poliprotein	185	EU218534	[383]
RT-qPCR (SYBR Green)		VP3	226	EF219380	[374]
		RdRP	137		
		ORF2	114		
			203	n.k.	[389]
			114	NC_009025	[368]
RT-qPCR (SYBR Green)		ORF2 RdRP	226 137	n.k.	[374]
Multi-point PCR (SYBR Green)		VP3 VP1 RdRP	298 225 219	KC690270	[387]
RT-qPCR (TaqMan)		RNApol	63	EU436450	[421]
Kashmir Bee Virus (KBV)					
RT-PCR		RdRP	417	NC_004807	[397]
			683	AY275710	[424]
		3Cpro	290		[361]
		ORF2	395		[388]
		capsid	625	NC004807	[420]
RT-qPCR (SYBR Green)		3Cpro	69	AY275710	[418]
			122		[376]
		ORF2	200		[389]
RT-qPCR (TaqMan)		RdRP	63	AY275710	[421]
		VP3	69	AF263725	[328]
Lake Sinai Virus (LSV)					
RT-PCR		capsid polyprotein	365	NC_032433	[369]
			205	NC_032433	[162]
	LSV1	RdRP	672	HQ871931	
	LSV2	capsid protein	558	HQ888865	
	LSV3	RdRP	243	JQ480620	[368]
	LSV4		379	JX878492	
	LSV5		190	KC880124	
RT-qPCR (SYBR Green)	LSV universal	RdRP	188	NC_032433	[162]
	LSV1		153	HQ871931	
	LSV2		225	HQ888865	
RT-qPCR (TaqMan)	LSV1, 2, 3, 4 universal	RdRP	152	n.k.	[368]
	LSV3		123	KY465717	[405]
Moku virus (MV)					
RT-qPCR (SYBR Green)		RdRP	93	KU645789	[365]
Slow Bee Paralysis Virus (SBPV)					
RT-qPCR (SYBR Green)		n.k.	226	NC_014137 KY243931	[389]
Sacbrood Virus (SBV)					
RT-PCR		helicase	823	AF092924	[351]
			123	AF092924	[368]
			824	AF092924	[334]
		SBV genome	variable		[425]
		5′UTR	487		[388]
		structural proteins	816		[426]
			211		[361]
		RdRP	426		[326]
		capsid	693		[420]
RT-qPCR (SYBR Green)		RdRP	70	NC002066	[418]
		VP3	335	AF092924	[389]
RT-qPCR (TaqMan)		polyprotein	70	AF092924	[363]
			106	MG545287	[405]
		RdRP	70	NC002066	[328]
		VP3	103	AF092924	[376]
ABPV, BQCV, CBPV, DWV, IAPV, SBV					
RT-PCR multiplex	ABPV	forward primer: intergenic region of Aparavirus (generic primer of both ABPV and IAPV); reverse primer: polyprotein	460	AF486073	[23]
	BQCV	polyprotein	536	EF517520/7762	
	CBPV	RdRP	774	EU122229	
	DWV	structural polyprotein	269	GU109335	
	IAPV	intergenic region/polyprotein	158	HQ897161	
	SBV	polyprotein	342	AF092924	
ABPV, BQCV, SBV					
RT-PCR multiplex	ABPV	ORF2	202	NC_002548	[22]
	BQCV	ORF1	322	AF183905: 379-700	
	SBV	ORF	487	NC_002066: 221-708	

n.k., not known.

## 3. Conclusions

In this article, we have presented an overview of available molecular diagnostics and genotyping tools for the detection and analysis of the main pathogens of the Western honeybee. This overview should assist in the selection of adequate tools for planned research and inform on a potential lack of molecular tools that would be important to be developed.

The available molecular tools are indispensable for: (i) in-depth investigations of still-understudied or emerging bee pathogens, (ii) detection of as-yet-unknown or cryptic species and elucidation of their strain composition, (iii) studies of coinfection with particular bee pathogens that synergistically increase pathogenicity, (iv) worldwide surveillance of the most virulent pathogens, (v) tracing the source of outbreaks, (v) a rapid response to emerging pathogens (e.g., *T. mercedesae*), and (vi) eradication programs or rapid and cost-efficient screening of hive products for pathogen contamination (e.g., *A. tumida*).

Furthermore, the presented information is aimed at facilitating an integrated view on the particularities of each pathogen and pathogenic group and interacting effects between them, as well as their commonalities. On the other hand, it allows for comparing and contrasting the molecular diagnostic approaches used to study these pathogens and pathogenic groups.

Many of the pathogenic agents dealt with in this review have been introduced into the managed population of the Western honeybee through man-made upheavals or by trading of bees. As evolutionary adaptation of honeybees to introduced alien pathogens is an extremely slow and gradual process, human intervention is needed to prevent their further distribution and to minimize their damage on the honeybee population. The availability of reliable diagnostic methods for the sensitive and specific detection of bee pathogens is the first step needed in the efficient implementation of control measures.

## Data Availability

Data is contained within the article or supplementary material.

## Supplementary Materials

The following supporting information can be downloaded at: https://www.mdpi.com/article/10.3390/vetsci9050221/s1, Table S1: Overview of selection criteria for the use and characteristics of different molecular diagnostic assays; Table S2: Criteria of selection of genotyping assays for strain differentiation of pathogens; Table S3: Number of scientific publications for each group and species of honeybee pathogens; Table S4: List of abbreviations.
