# Supplementary material for: Molecular Detection and Differentiation of Arthropod, Fungal, Protozoan, Bacterial and Viral Pathogens of Honeybees
Source: Vet Sci. 2022 May 2;9(5):221. doi: 10.3390/vetsci9050221 (PMC9145064; doi:10.3390/vetsci9050221)
Supplement: Supplementary file 1 [file vetsci-09-00221-s001.zip › Table S1 Diagnostics.pdf]

Tabla S1 Overview of selection criteria for the use and characteristics of different molecular diagnostic assays[a]

	**PCR (RT-PCR)**	**multiplex PCR**	**PCR-RFLP**	**qPCR (RT-qPCR)**	**multiplex qPCR**	**LAMP**	**nPCR**	**snPCR[b]**	**HRM**
Specificity	+	+	++	+++	+++	+++	+++	++	+++
Sensitivity	+	+	+	+++	+++	+++	+++	++	+++
Reproducibility	++	++	++	+++	+++	++	++	++	+++
Pathogen quantification	no	no	no	yes	yes	no	no	no	no
Difficulty of result interpretation	+	++	+++	++	++	+	+	+	++
Expertise needed	+	+	++	+++	+++	+	++	++	+++
Development efforts	+	++	++	+++	+++	+++	++	++	+++
Equipment needed	++	++	++	+++	+++	+	++	++	+++
Cost per sample	+	++	++	++	+++	+	++	++	++

[a]Indications are for guidance only and don not substitute a thorough planning of a study; [b]snPCR, seminested PCR
