# Supplementary material for: Molecular Detection and Differentiation of Arthropod, Fungal, Protozoan, Bacterial and Viral Pathogens of Honeybees
Source: Vet Sci. 2022 May 2;9(5):221. doi: 10.3390/vetsci9050221 (PMC9145064; doi:10.3390/vetsci9050221)
Supplement: Supplementary file 1 [file vetsci-09-00221-s001.zip › Table S2 Genotyping.pdf]

Table S2 Criteria of selection of genotyping assays for strain differentiation of pathogens[a]

Variables critical for application	Genotyping assays									
	Fingerprinting (detection of genome polymorphism or clonality)			Codominant markers (detection of allele polymorphism)						
	**RAPD**	**MLVA**	**rep-PCR**	**PCR-RFLP**	**ARMS-PCR**	**[b]HRM**	**micro-satellites**	**MLST**	**cgMLST/ wgMLST**	**wgSNP**
Strain isolation required	yes	yes	yes	no	no	no	no	yes	yes	yes
Reproducibility	low	medium	high	very high	very high	high	very high	very high	very high	very high
Power of strain discrimination (or resolution of polymorphism)	medium	medium	medium	low	low	medium	high	high	very high/ maximal	maximal
Suitable for building reference database[c]	no	yes	yes	n.a.	n.a.	n.a.	no	yes	yes	(yes)
Separation of outbreak strain clusters	no	[d](yes)	no	no	no	no	[d]((yes))	[d](yes)	yes	yes
Expertise required	low	medium	medium	low	low	high	high	high	very high	very high
Developmental cost	low	medium	medium	low	low	medium	high	high	very high	very high
Cost per sample	low	low	low	low	low	medium	medium	medium	high	high

[a]Indications are for guidance only and do not substitute a thorough planning of a study; [b]high resolution melting analysis; [c]by compilation of defined sequence types or fingerprint variants; [d]brackets indicate that ad hoc studies might occasionally resolve the strain identity but this is not guaranteed by this methods since their resolution is either limited or sequence types cannot be defined; n.a., not applicable
