# Supplementary material for: Molecular Detection and Differentiation of Arthropod, Fungal, Protozoan, Bacterial and Viral Pathogens of Honeybees
Source: Vet Sci. 2022 May 2;9(5):221. doi: 10.3390/vetsci9050221 (PMC9145064; doi:10.3390/vetsci9050221)
Supplement: Supplementary file 1 [file vetsci-09-00221-s001.zip › Table S3 Publications.pdf]

Table S3 Amount of scientific publications for each group and species of honey bee pathogens. The total number of publications from 1979 to January 2022 (Total) is given. The number of publicationes released in 2021 and the average number of publications in the last 5 years are shown as an indication of the actual and recent interest on these pathogens, respectively. Searches were carried out in Scopus® using "*Apis mellifera*" and the corresponding genus as keywords, e.g. "*Varroa*". All bee-infecting virus are considered together. A search using "Varroa AND NOT virus" was also performed, to get an overview of the scientific attention on the arthropod itself, not as a viral vector.

Kingdom	Genus	Total	2021	Average 2017-2021
Arthropoda	*Varroa*	1433	89	69
	Aethina	164	6	6
	Acarapis	104	2	2
	Tropilaelaps	52	4	3
Virus	-	704	59	46
Fungi	*Nosema*	566	45	36
	Ascosphaera	114	3	5
	Aspergillus	37	6	2
Bacteria	*Paenibacillus*	302	14	14
	Melissococcus	46	1	3
	Serratia	20	2	2
	Spiroplasma	18	0	1
Protozoa	*Crithidia*	28	5	3
	Lotmaria	12	4	2
	Apicystis	6	2	1
	Malpighamoeba	4	0	0
	TOTAL	3610	242	195
	Varroa AND NOT Virus	1179	68	55
