# Supplementary material for: Molecular Detection and Differentiation of Arthropod, Fungal, Protozoan, Bacterial and Viral Pathogens of Honeybees
Source: Vet Sci. 2022 May 2;9(5):221. doi: 10.3390/vetsci9050221 (PMC9145064; doi:10.3390/vetsci9050221)
Supplement: Supplementary file 1 [file vetsci-09-00221-s001.zip › Table S4 Abreviations.pdf]

List of Abbreviations

ABPV	Acute bee paralysis virus
AFB	American Foulbrood
ALPV	Aphid lethal paralysis virus
AmFV	*Apis mellifera* filamentous virus
ARMS-PCR	Amplification-refractory mutation system-PCR
ARV	*Apis mellifera* rhabdovirus
atp6	*ATP synthase subunit 6*
Bar-HRM	Barcode-high resolution melting analysis
BeeMLV	Bee macula like virus
BQCV	Black queen cell virus
CBPV	Chronic bee paralysis virus
CC	clonal complexes
CCD	Colony Collapse Disorder
cgMLST	Core genome MLST
cox1 - cox3	*Cytochrome c oxidase subunit 1 - 3*
cytb	*Cytochrome b*
DGGE-PCR	Denaturing-gradient gel electrophoresis
DWV	Deformed wing virus
EFB	European Foulbrood
ERIC	Enterobacterial repetitive intergenic consensus
Fur	Ferric uptake regulator family
GAPDH	Glyceraldehyde 3-phosphate dehydrogenase gene
gyrB	DNA gyrase subunit B gene
HRM	High resolution melting analysis
IAPV	Israeli acute paralysis virus
IIV	Iridescent invertebrate virus
IRES	Internal ribosome entry site
ItuC	Iturinic lipopeptide subunit C
KBV	Kashmer bee virus
KV	Kakugo virus
LAMP	Loop mediated isothermal amplification
LSV	Lake Sinaí virus
MLST	Multi-locus sequence typing
MLVA	Multiple locus variable number of tandem repeat analysis
msa	Multiple sequence alignment
MV	Moku virus
napA	Na+/H+ antiporter gene
NaVCh	Voltage-gated sodium channel gene
nd4/ nad5	NADH dehydrogenase subunit 4 - 5 gene
OIE	International Office of Epizooties
ORF	Open reading frame
PCR-RFLP	PCR-restriction-fragment length-polymorphism
PCR-SSPC	Single-strand conformation-polymorphism analysis after PCR-amplification
plx1	*Paenibacillus larvae* toxin 1
qPCR	Quantitative PCR
RAPD	Randomly-amplified polymorphic DNA
RdRp	RNA-dependent RNA polymerase gene
REP	Enterobacterial repetitive extragenic palindromic

rep-PCR	Repetitive element sequence-based PCR
rpb1	DNA-directed RNA polymerase II subunit gene
rpoB	RNA polymerase beta subunit gene
RT-PCR	Reverse transcription PCR
RT-qPCR	Reverse transcription qPCR
SBPV	Slow bee paralysis virus
SBV	Sacbrood bee virus
SHB	Small hive beetle
SNP	Single nucleotide polymorphism
sodA	Manganese-dependent superoxide dismutase gene
ssRNA	Single string RNA
ST	Sequence types
TBM	Thoracic disc method
tnp60	Putative transposase gene
$tRNA^{cys}$	tRNA **gene** for cysteine
VDV-1/DWV-B	*Varroa destructor* virus 1/deformed wing virus B
VL	Viral load
VNTR	Variable number of tandem repeats
VOV	Varroa orthomyxovirus
VP	Virus portein
wgMLST	Whole genome MLST
WGS	Whole-genome sequence
wgSNP	Single-nucleotide polymorphism of a query isolate genome
